# Transcriptome analysis reveals the neuroprotective effect of Dlg4 against fastigial nucleus stimulation-induced ischemia/reperfusion injury in rats

**DOI:** 10.1186/s12868-023-00811-6

**Published:** 2023-07-31

**Authors:** Jinggui Gao, Xiaomin Pang, Lei Zhang, Shenghua Li, Zhenxiu Qin, Xiaoyun Xie, Jingli Liu

**Affiliations:** Department of Neurology, The First Affiliated Hospital, Guangxi Medical University, Nanning, China

**Keywords:** Fastigial nucleus stimulation, Dlg4, Neuroprotective, Transcriptome analysis, Ischemia, Alternative polyadenylation (APA)

## Abstract

**Background:**

Previous studies have demonstrated that electrical stimulation of the cerebellar fastigial nucleus (FNS) can considerably decrease infarction volume and improve neurofunction restoration following cerebral ischemia. Nevertheless, the molecular mechanism of the neuroprotective effect of FNS is still vague.

**Methods:**

In this study, we developed a rat model of ischemia/reperfusion that included 1 h FNS followed by reperfusion for 3, 6, 12, 24, and 72 h. The expression profile of molecular alterations in brain tissues was obtained by transcriptome sequencing at five different time points. The function and pathway of miRNA expression pattern and core genes were annotated by Allen Brain Atlas, STRING database and Cytoscape software, so as to explore the mechanism of FNS-mediated neuroprotection.

**Results:**

The results indicated that FNS is associated with the neurotransmitter cycle pathway. FNS may regulate the release of monoamine neurotransmitters in synaptic vesicles by targeting the corresponding miRNAs through core Dlg4 gene, stimulate the Alternative polyadenylation (APA) incident’s anti -apoptosis effect on the brain, and stimulate the interaction activation of neurons in cerebellum, cortex/thalamus and other brain regions, regulate neurovascular coupling, and reduce cerebral damage.

**Conclusion:**

FNS may activate neuronal and neurovascular coupling by regulating the release of neurotransmitters in synaptic vesicles through the methylation of core Dlg4 gene and the corresponding transcription factors and protein kinases, inducing the anti-apoptotic mechanism of APA events. The findings from our investigation offer a new perspective on the way brain tissue responds to FNS-driven neuroprotection.

**Supplementary Information:**

The online version contains supplementary material available at 10.1186/s12868-023-00811-6.

## Backgrounds

Ischemic stroke is one of the main causes of permanent disability; however, the available treatment options are limited [1]. Energy exhaustion, excitatory poisoning, Calcium overload, neuroinflammation, apoptosis, and oxidative stress are involved in the pathological mechanism of ischemic injury [2–6]. Therefore, interventions aimed at reducing ischemic injury are attractive new therapeutic strategies for ischemic stroke. Fastigial nucleus stimulation (FNS) is an experimental treatment that promotes the recovery of neurological function in vitro and has a neuroprotective effect [7]; however, specific mechanism of the effect is unknown. Recent studies have shown that 1 h FNS can reduce the volume of focal ischemic infarction in rats by nearly 40%, protect the neurons from apoptosis, and reduce cerebrovascular inflammation [8, 9]. FNS-induced neuroprotection lasts for up to 30 days [10].

Despite the wide array of protective benefits FNS contributes, its most distinct advantage involves the immediate activation of neural pathways between disparate brain regions through electrical stimulation, setting it apart from numerous other neuroprotective therapies [11, 12]. Systemic administration of drugs depends on cerebral blood flow and the ability to pass through the blood-brain barrier to reach the target brain region. However, during acute cerebral ischemia, cerebral blood flow is often blocked, and drugs cannot be efficiently delivered to the damaged tissue. In contrast, during electrical stimulation, the current reaches the target brain region directly, and FNS delivery does not depend on cerebral blood flow or the state of the blood-brain barrier [13, 14]. miRNAs are important negative regulators of gene expression and can regulate a variety of biological pathways. miRNAs are regulated during cerebral prestimulation and can enhance neuroprotection [15–17]. However, little is known about the protective effect of miRNAs on FNS-induced brain injury, and there is no information about the continuous changes in the brain transcriptome induced by FNS-mediated neuroprotective effects. Therefore, our study aimed to create a database of miRNAs expressed in the brains of rats subjected to cerebral ischemia/reperfusion for 3, 6, 12, 24, or 72 h after 1 h FNS to determine a potential mechanism of the neuroprotective effect of FNS and miRNAs in brain injury.

Neurons in the cerebellum, like other brain regions, are known to interact by changing their firing rates and firing at different rhythms [18]. By selectively altering their emission patterns, cerebellar neurons can distribute novel information more efficiently than if they uniformly fired at a constant rate [19]. By combining the information of Allen Brain Atlas database, we explored the neural electrical signal communication between the cerebellar parietal nucleus and the hypothalamus, cortex and other brain regions [20, 21].

## Methods and materials

### Research and design

**Fig. 1:**
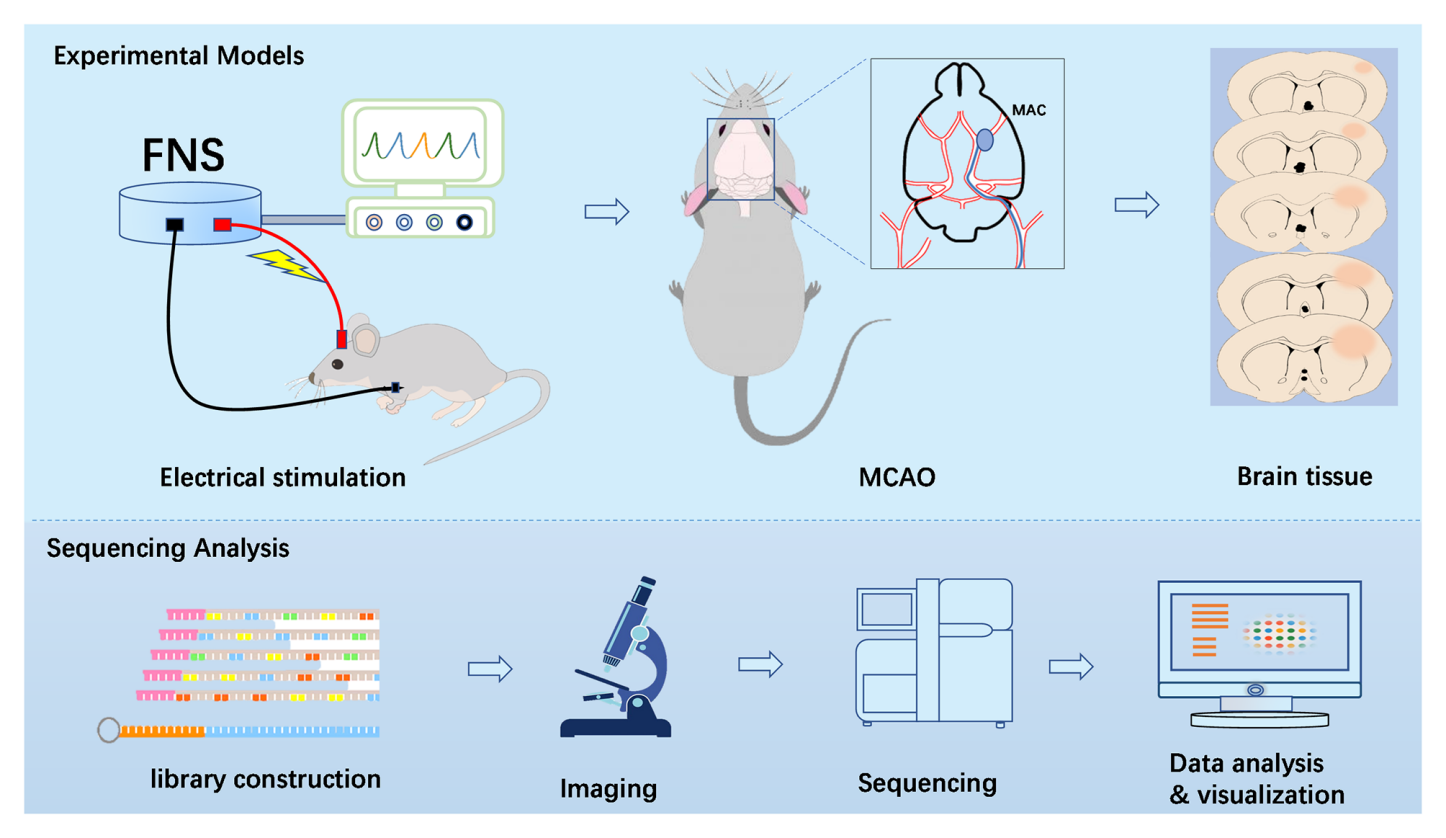
Flow chart of the experimental design. (Created with BioRender.com)

The study included two steps. The first step included the development of an experimental model, and the second step included the acquisition of the brain tissue samples for transcriptome sequencing. In the experimental modeling stage, we used an electrical stimulation device to stimulate the rats for 1 h and then generated a rat model of ischemia/reperfusion. Ischemia/reperfusion was performed for 3, 6, 12, 24, or 72 h (5 rats at each time point), and the animals of the control groups (5 rats at each time point) were not subjected to FNS. Then, brain tissues were collected during the second step for sequencing analysis. Finally, the sequencing results were comprehensively assessed and visually analyzed (Fig. 1).

### Animals

The Experimental Animal Center of Guangxi Medical University supplied pathogen-free adult male Sprague-Dawley rats weighing 280-300 g. A total of 59 rats were used in the study. The data of 50 rats were reported. Nine rats were excluded from the study, including five that had no neurological defects and four that died during the experiment. The rats were randomly divided into two groups: (1) the I/R control group (right middle cerebral artery occlusion for 2 h followed by reperfusion for 3, 6, 12, 24, and 72 h; 5 rats at each time point) and (2) I/R + FNS group (electrical stimulation of the right cerebellar fastigial nucleus for 1 h, subsequent cerebral ischemia for 2 h, and reperfusion for 3, 6, 12, 24, and 72 h; 5 rats at each time point). Animals were handled according to the guidelines of the Council of the International Organization for Animal Experimental Medicine (World Health Organization, Geneva, Switzerland). The Animal Care and use Committee of Guangxi Medical University approved the animal protocol (No.202,005,009).

### Cerebellar Fastigial Nucleus Stimulation (FNS)

Before the procedure, the rats were subjected to an 8–12 h fasting period. The rats were anesthetized with isoflurane gas inhalation and placed on a brain stereotaxic instrument. A median incision at the top of the skull exposed the skull, and the periosteum was removed. The location of the cerebellar fastigial nucleus was determined according to the stereotaxic map of the brain of Wistar rats. The position 11.1 mm posterior of the posterior edge of the anterior fontanelle was used as stereotaxic zero, and the midline was opened 1.1 mm to the left. A hole was drilled into the skull, and a stimulation needle was inserted to a depth of approximately 5.6 mm. A software-controlled YC-2 stimulator (Chengdu Instrument Factory) was used for electrical stimulation, the range of stimulus parameters reported in the previous literature was used for setting [8, 22–25]: current intensity 70 µA, DC square wave pulse, frequency 50 Hz, and time history 0.5 ms. The time of each stimulation was 1 h. Under this parameter of electrical stimulation frequency, stimulation of neurons can play a good protective role. All rats were stimulated under superficial anesthesia. After the stimulation, the needle was removed, and the scalp was sutured.

### Middle cerebral artery occlusion and reperfusion (MCAO/R)

Rat model of cerebral ischemia/reperfusion (I/R) was generated based on the method of Longa. The rats were anesthetized with isoflurane gas inhalation and fixed in the supine position on an operating table. After disinfection, an incision was made from the middle of the neck, and the anterior cervical muscle was bluntly separated to expose the right common carotid artery, external carotid artery, and internal carotid artery. The proximal end of the common carotid artery and the proximal end of the external carotid artery were ligated with an arterial clamp to temporarily block the blood flow of the internal carotid artery. A small opening at the junction of the external carotid artery near the common carotid artery was cut to insert a nylon thread bolt, and the artery clamps were released. The nylon line was gently pushed into the right middle cerebral artery to the depth of (18.5 ± 0.5) mm at the bifurcation to block the blood flow of the right middle cerebral artery. After operation, the skin was sutured to leave 1 cm of the nylon thread on the outside. After 2 h of ischemia, the rats were reperfused by gently lifting the outside end of the nylon thread leading to the stump of the external carotid artery to restore the blood flow. The rats were reperfused for 3, 6, 12, 24, and 72 h.

### Measurement of the cerebral infarction volume

The volume of cerebral infarction was determined by the triphenyltetrazolium chloride (TTC) staining. After 3, 6, 12, 24, and 72 h of ischemia/reperfusion, the rats were anesthetized with 2% sodium pentobarbital (0.3 mL/100 g) i.p and then underwent quick decapitation; the brain was carefully removed from the skull and kept at -20 °C for 30 min. The frozen brain was cut into continuous 2 mm coronal sections and immersed in 2% TTC solution at 37 °C for 30 min. The infarct volume of each brain section was analyzed by a Luxex-F image analyzer.

### Preparation and sequencing of the miRNA Library

After ischemia/reperfusion, the brain was dissected to harvest the tissue from the ipsilateral cortex beyond optic nerve chiasm. RNA was extracted with TRIzol, and the RNA concentration was detected by using a NanoDrop spectrophotometer (ND2000, Thermo Fisher Scientific, Waltham, MA, USA). The expression levels of various microRNAs were determined by using an Illumina HiSeq 2500 platform by LC-BIO (Hangzhou, China). The cDNA ends were repaired by agarose gel purification, and adenine was added to the 3’-ends of the cDNA followed by ligation. PCR was used to determine the size and amplify the fragments. Finally, single-end sequencing was performed by using an Illumina Truseq SmallRNA preparation kit (Illumina, San Diego, California, USA).

### miRNA analysis

Data analysis and filtering, including low-quality sequence, linker, sequence length, and copy number filtering were performed using acgt101-mirv4.2 sequencing data analysis software by LC Sciences. After conservative analysis of miRNAs and comparison with the SangermiRBase 17.0 database, the background values were subtracted, and signal was normalized using a Lowess filter to exclude the errors caused by inconsistencies in the sample content and fluorescent labeling. Finally, pairwise comparison between the groups was used to identify differentially expressed miRNAs (DEMs) using DESeq (P < 0.05 and |log_2_fold change| > 1) (Table S1).

### Neural circuit analysis of cerebellar fastigial nucleus

The gene regulation functions of miRNAs primarily involve complementary sequencing with target gene transcripts. Therefore, it is crucial to understand the detailed function of the miRNA target genes (MTGs). We used miRWalk [26] (http://mirwalk.umm.uniheidelberg.de/) and DIANA software [27] (http://diana.imis.athena-innovation.gr/DianaTools/index.php) to predict the target genes of the sequenced miRNAs and selected the targets predicted by both databases. These genes were defined as important target genes. Furthermore, we utilized the web-based gene set analysis toolkit (WebGestalt) database [28] (http://www.webgestalt.org/) and STRING database [29] (https://string-db.org/) to perform GO and KEGG pathway enrichment analyses on these targets, determining important pathophysiological genes and pathways.

### Enrichment analysis of transcription factors and kinases

eXpression2Kinases [30] (X2K) is a reliable systems biology tool for biomedical studies that can be used to constructed the upstream cell signal networks of miRNAs by connecting the expression characteristics of the target genes with the upstream genes. Kinase enrichment analysis (KEA) was used to identify the substrates of phosphorylated transcription factors and protein kinases that connect the intermediate proteins. Additionally, we assessed the expression of transcription factors in the brain using Harmonizome [31] (http://amp.pharm.mssm.edu/Harmonizome/), which is a collection of genes, proteins, and their functions.

### Core gene and methylation analysis

In this study, the STRING database of gene interactions was used to construct a gene interaction network. We selected the targets, which have been validated by experimental evidence and have a high degree of confidence in their relationships, as inputs for Cytoscape v3.7.0. Then, the CytoHubba plug-in [32] was used to identify the core genes according to the degree algorithm, and the blood brain DNA methylation comparison tool [33] (https://epigenetics.essex.ac.uk/bloodbrain/) was used to perform methylation analysis of the core genes. We analyzed the methylation of the core Dlg4 gene using samples from blood and four brain regions (prefrontal cortex, entorhinal cortex, superior temporal gyrus, and cerebellum).Then, the PhenoGen database [34] (https://phenogen.org/), which can be used to investigate the qualitative characteristics of a single gene, was used to perform the expression quantitative trait loci (eQTL) analysis to identify the chromosomal regions corresponding to the location of the gene expression trait locus. Finally, the Harmonizome database was used to perform the ontology analysis of the core genes, and receiver operating characteristic (ROC) curve analysis was used to evaluate the accuracy of the results.

### Alternative polyadenylation (APA) event analysis of Dlg4

Alternative polyadenylation (APA) is a molecular mechanism during pre-mRNA processing that involves the use of multiple polyadenylation sites (PA sites) to generate transcripts of lengths from a single gene, expanding the mRNA transcript subtype diversity. Dysregulation of APA can disturb gene expression and induce protein dysfunction, which is associated with many diseases including neuronal degeneration in the brain [35]. APA is now recognized as an important regulatory mechanism in the pathophysiology of human disease [36]. Here, we analyzed the APA profile of Dlg4 using the APAatlas database and analyzed the association between APA events and traits and mRNA expression. These APA events may reveal novel mechanisms for transcriptional regulation, tissue development, and phenotype, which we linked APA to disease genes to explore the mechanism of APA events after stroke [37].

### Statistical analysis

Statistical analysis was performed using SPSS21.0 software. The data are expressed as the mean ± standard deviation (SD). T-test was used for comparison of two groups. ***P*** < 0.05 was considered statistically significant.

## Results

### FNS reduces the infarct volume in Ischemia/Reperfusion

**Fig. 2:**
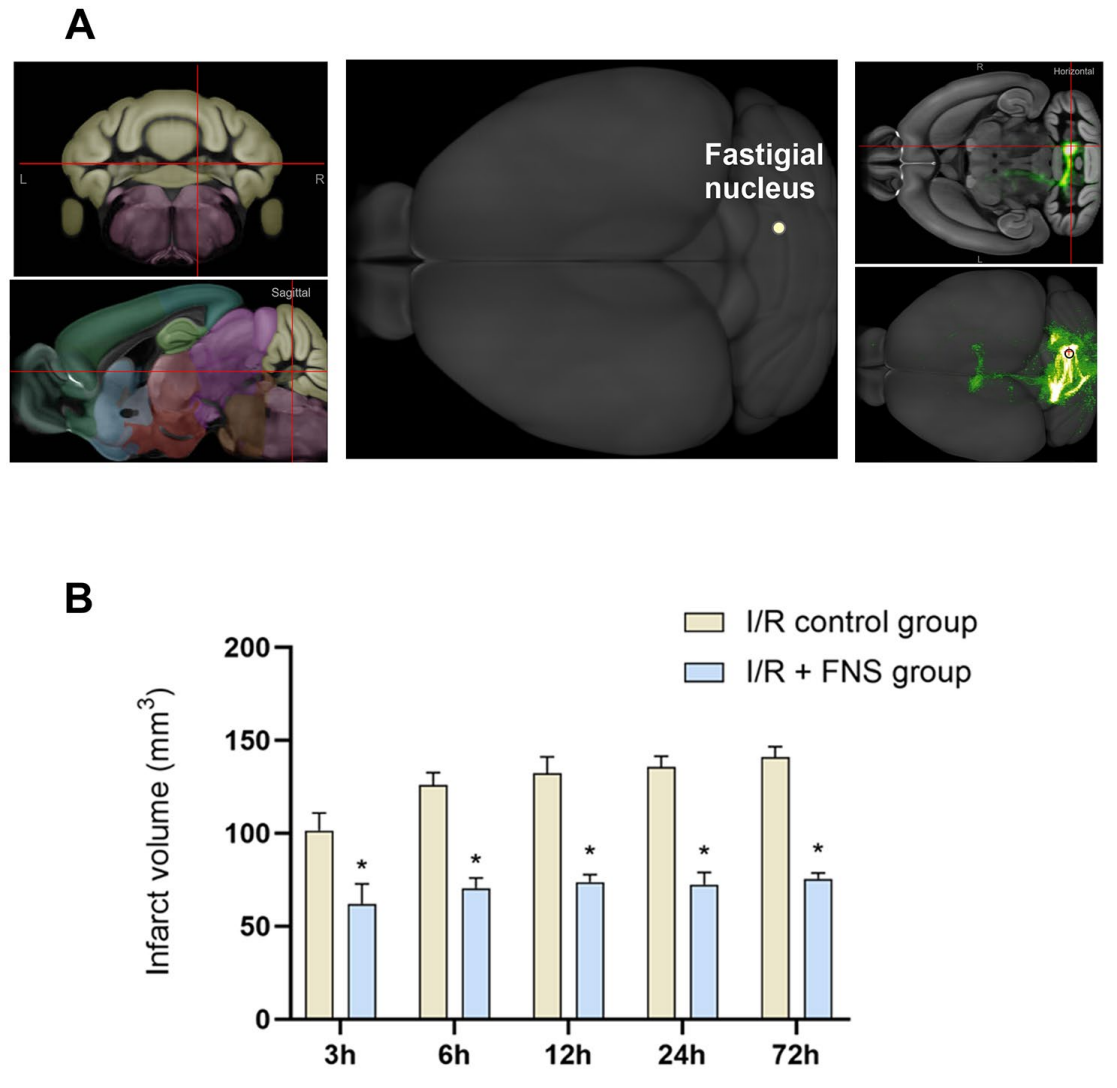
FNS Reduces the Infarct Volume in Ischemia/Reperfusion. **A** Mapping of different dimensions of the cerebellar fastigial nucleus. **B** The cerebral infarction volume in rats in the I/R + FNS group was significantly lower than that in the I/R control group by approximately 43.5% at all time points. ***P***<0.05

We used Allen Brain Atlas to determine the three-dimensional localization of the cerebellar parietal nucleus. To investigate the neuroprotective effect of FNS on I/R injury, we compared the infarct volume in the experimental and control groups. The volume of cerebral ischemic infarction was significantly reduced by approximately 40% at various time points in the I/R + FNS group compared with that in the I/R control group (***P*** < 0.05) (Fig. 2).

### Bioinformatics Analysis of DEMs

**Fig. 3:**
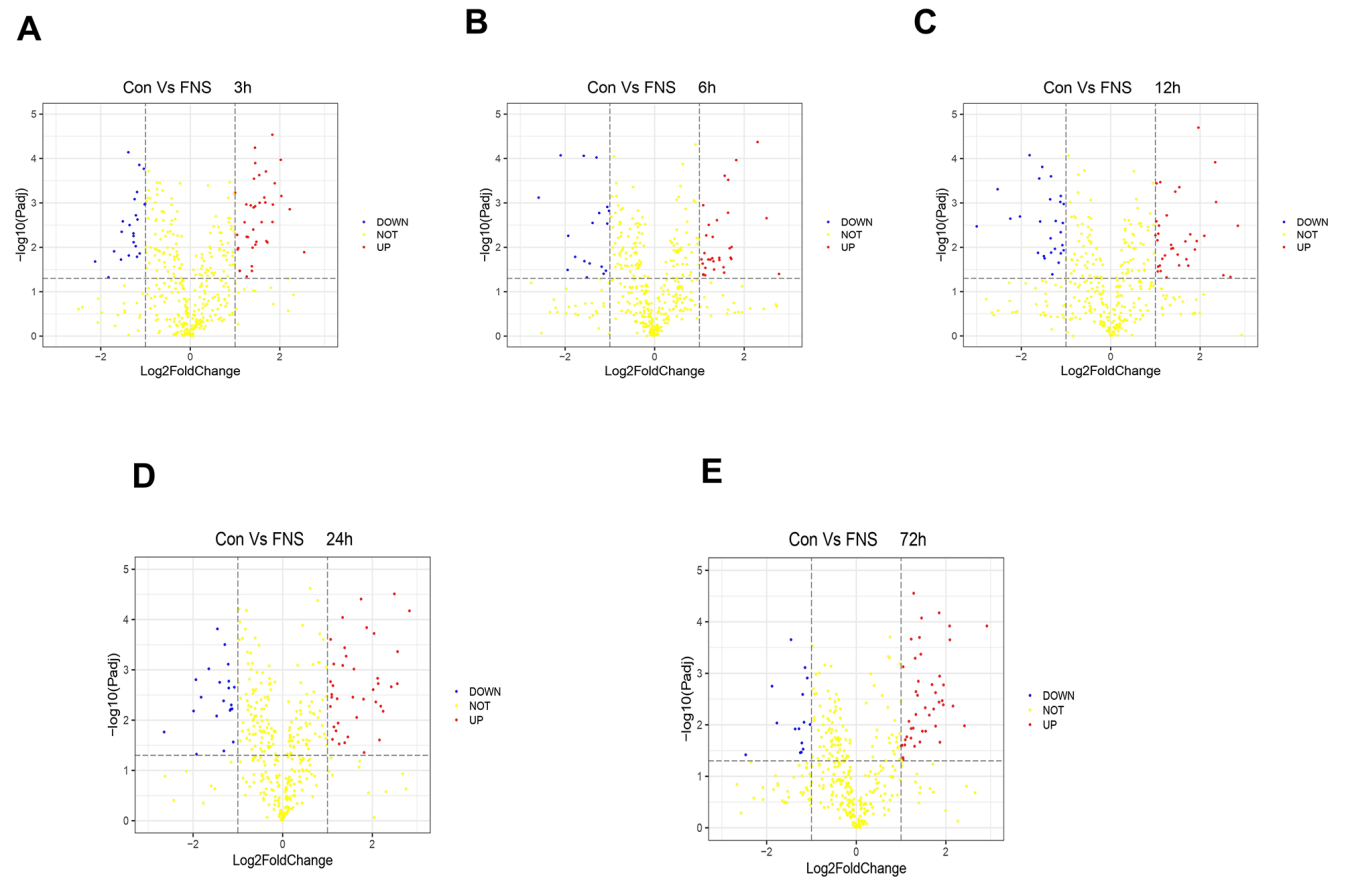
Volcanic map of DEMs. (**A-E**) Panels A, B, C, D, and E show the miRNA expression profiles in the IR + FNS group and the control IR group at 3, 6, 12, 24, and 72 h, respectively. Red and blue represent upregulated and downregulated miRNAs, respectively

According to the experimental grouping, a visual volcano map (Fig. 3) was constructed based on the sequencing results at 3, 6, 12, 24, and 72 h to illustrate the DEMs at various time points. The data of the volcanic and cluster analysis maps indicate that the miRNA expression profile of the IR + FNS group was significantly different from that of the control group at five time points and that the trend is consistent suggesting that the effect of FNS on rat ischemic brain tissue is relatively stable and that a specific neuroprotective regulatory mechanism may be involved in this effect.

### Neural circuit analysis

To predict target genes that were expressed at the same time point, we selected miRNAs with the same co-expression to predict target genes expressed trend. Then, target genes and functional pathways associated with these microRNAs and differentially regulated by FNS were identified. DIANA and miRWalk databases were used to predict target genes. Select overlapping target genes to increase prediction reliability. Ultimately, we screened a total of 2,187 target genes, indicating that miRNAs differentially regulated by FNS have broad impacts on gene regulation.

**Fig. 4:**
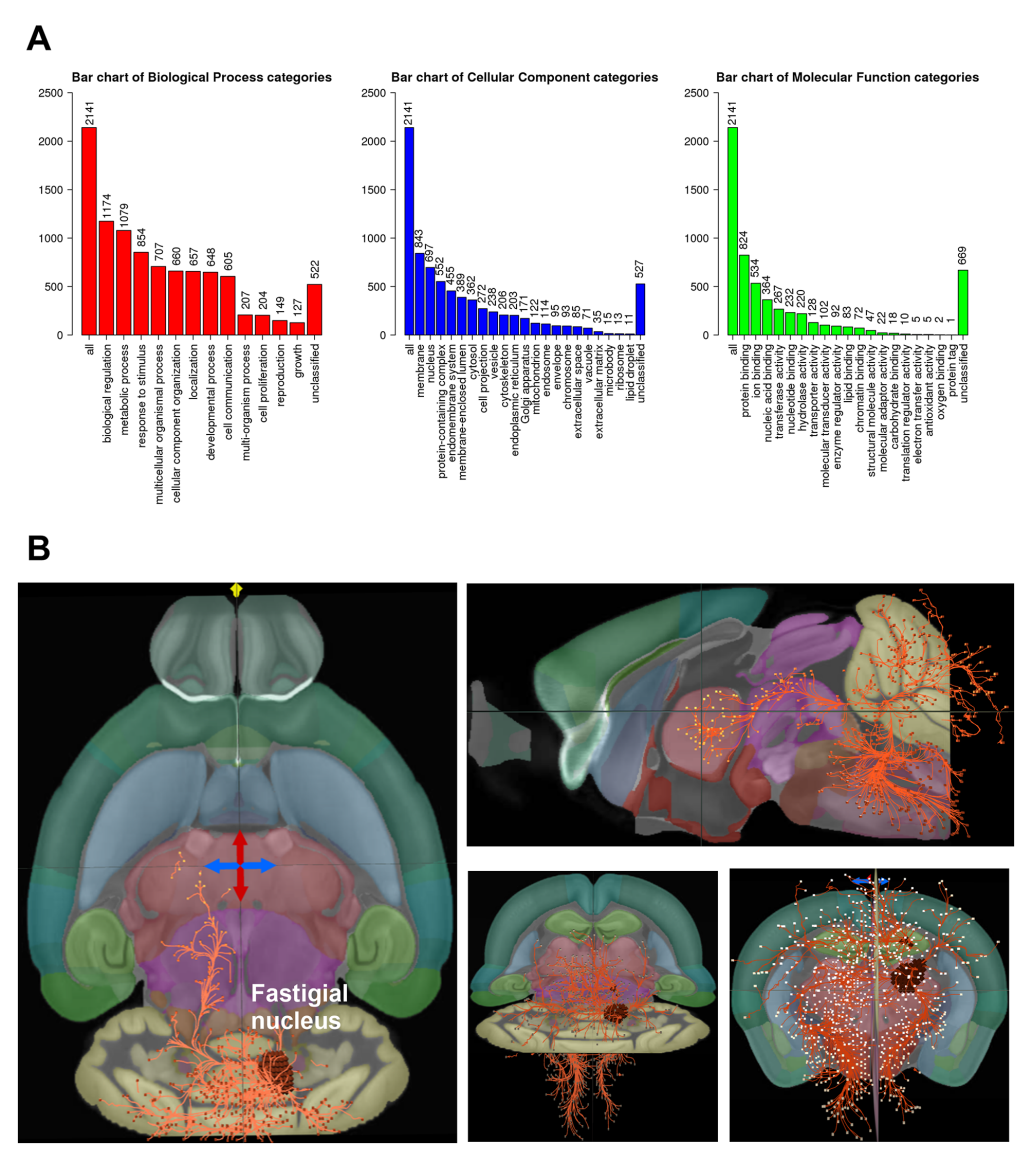
GO enrichment analysis and neural circuits projection analysis of target genes. **A** GO enrichment analysis of target genes. **B** Neural circuits in the cerebellar fastigial nucleus that projects to the brain

To investigate the details of the biological mechanisms of the influence of differentially expressed MTGs, Webgestalt was used to analyze MTGs, including biological process (BP), cellular component (CC), and molecular function (MF) (Fig. 4A). The results of MF analysis of MTGs showed that most of these genes have the functions of protein binding, ion binding, and nucleic acid binding. GO cellular components analysis showed that these differentially expressed genes (DEGs) are mainly concentrated on the surface of the cell membrane. GO BP analysis showed that these genes are mainly involved in metabolic processes and responses to stimuli. A total of 854 genes, which accounted for one-third of the total target genes, were involved in the response to stimulation indicating that FNS may regulate the target genes via miRNAs. MTGs were analyzed by KEGG using STRING, and the data indicated that MTGs are enriched in the neuronal system and neurotransmitter cycle pathways (Table 1). Six of the top 10 pathways were related to nerves, three pathways suggested that the targets are related to neurotransmitters, including the release of glutamate, serotonin, and dopamine, and one pathway was involved in cross chemical synaptic transmission. The KEGG analysis showed that a set of the genes is related to a variety of synaptic signals suggesting that these target genes may be involved in the regulation of neurons by FNS. We then used data from the Allen Brain Atlas to show the brain’s neural circuits projected by the parietal nucleus in 3D model (Fig. 4B). These data suggest that FNS may exert neuroprotective effects by activating different circuits and neurons in brain regions such as the hypothalamus, affecting the regulation of key neurotransmitters in the nervous system such as glutamate [24, 38–42].

**Table 1:** Pathway enrichment analysis of target genes

term	description	FDR
RNO-112316	Neuronal System	0.0011
RNO-199991	Membrane Trafficking	0.0011
RNO-5653656	Vesicle-mediated transport	0.0012
RNO-112315	Transmission across Chemical Synapses	0.0075
RNO-210500	Glutamate Neurotransmitter Release Cycle	0.0127
RNO-597592	Post-translational protein modification	0.0159
RNO-181429	Serotonin Neurotransmitter Release Cycle	0.0159
RNO-392499	Metabolism of proteins	0.0162
RNO-112310	Neurotransmitter release cycle	0.0231
RNO-212676	Dopamine Neurotransmitter Release Cycle	0.0232

### Analysis of transcription factors, kinases and interactions between MTGs

**Fig. 5:**
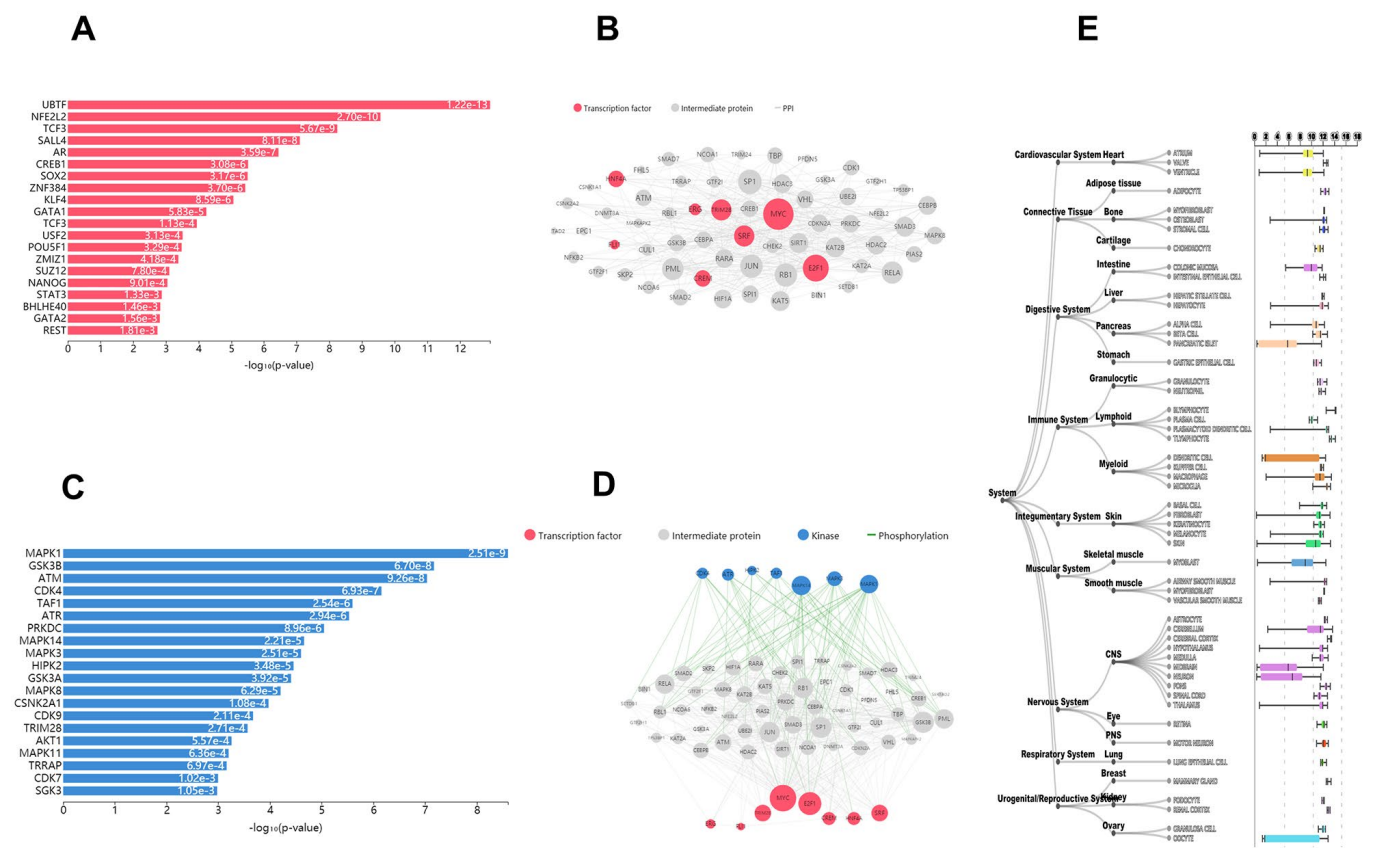
Analysis of the upstream transcription factors and protein kinases and construction of network interactions. **A** Analysis of the top 10 transcription factors. **B** Transcription factor and gene network interaction diagram. **C** Analysis of the top 10 kinases. **D** Transcription factor-gene-kinase X2K network map. **E** Dlg4 expression in tissues

We identified the transcription factors and kinases of genes that play a role in neuronal regulation. The top 10 transcription factors were UBTF, NFE2L2, TCF3, SALL4, AR, CREB1, SOX2, ZNF384, KLF4, and GATA1 (Fig. 5A), with UBTF showing the highest enrichment of all transcription factors. A protein encoded by UBTF plays a key role in an important aspect of ribosomal RNA transcription, including mediation of polymerase I recruitment to the promoter region of rDNA by RNA. UBTF can influence a number of synapses in neurons [43]. Furthermore, Harmonizome showed that UBTF is widely expressed in the central nervous system and is expressed highly in the cerebellum (Fig. 5E). The results of the protein kinase enrichment analysis identified the top 10 genes: MAPK1, CK2ALPHA, CSNK2A1, MAPK14, CDK4, ERK1, MAPK3, CDK1, HIPK2, and AKT1 (Fig. 5C). Finally, we utilized the X2K algorithm to simulate known protein-protein interactions and connect enriched transcription factors with kinases to generate a complete upstream pathway (Fig. 5B and D).

### Analysis of the Core genes by the STRING database and Cytoscape Software

**Fig. 6:**
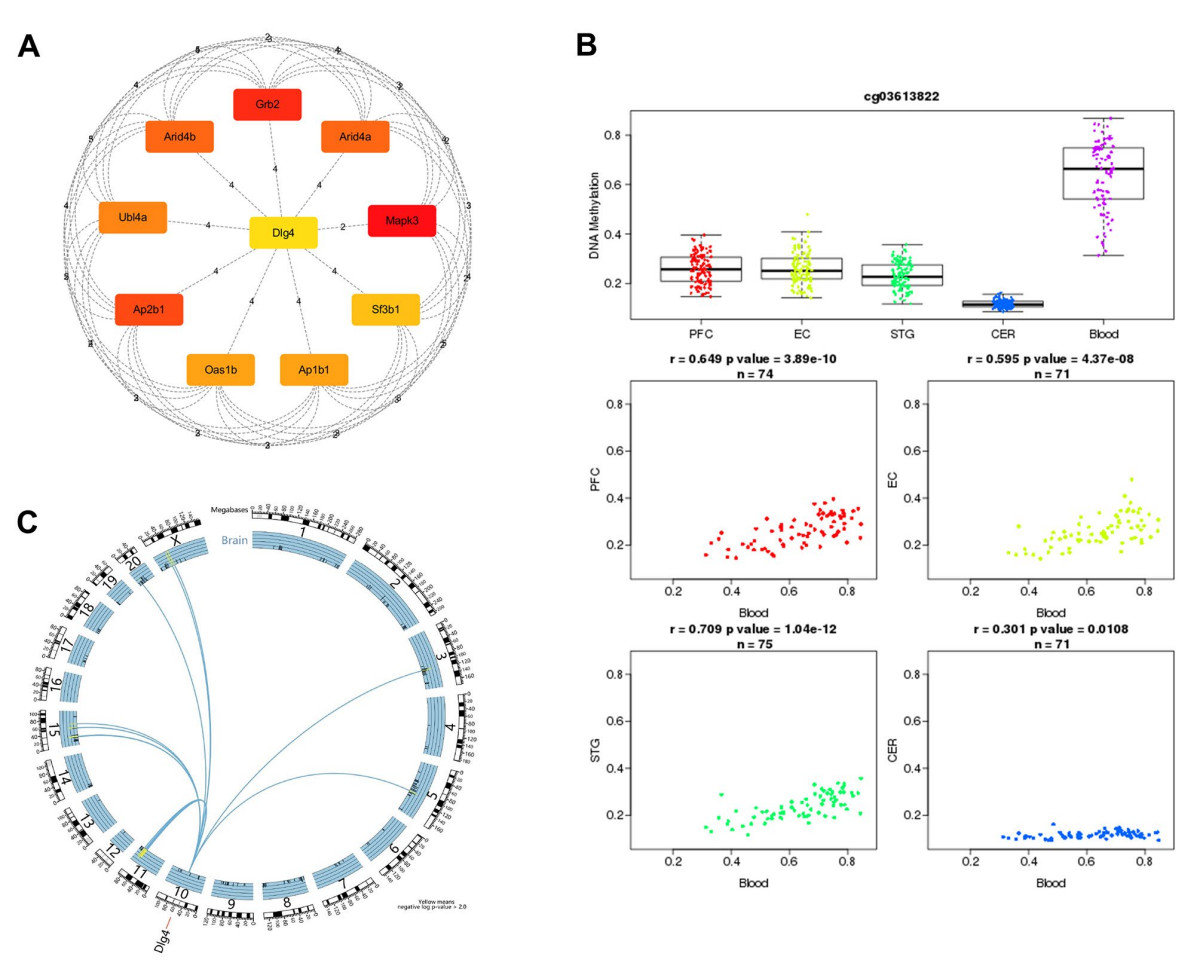
Methylation and DNA sequence analysis of the core Dlg4 gene. **A** Map of the top 10 core genes. **B** A box diagram of the distribution of DNA methylation divided by the Dlg4 levels in four regions of the brain and four scatter plots of the blood samples. **C** DNA sequences of rat chromosomes that are associated with the Dlg4 phenotype

The following core genes were identified by the analysis of the PPI network: Dlg4, Mapk3, Grb2, Ap2b1, Arid4a, Arid4b, Ubl4a, Ap1b1, Oas1b, and Sf3b1 (Fig. 6A). The map shows that Dlg4 is located in the center of the core genes indicating that Dlg4 is associated with each gene and has the highest number of target relationships. Dlg4 is a key synaptic protein for neuronal plasticity in the brain. The epigenetic mechanism of DNA methylation is one of the key factors of nerve development and function in the brain; however, the epigenetic mechanism of Dlg4 regulation of transcription is unknown. Hence, we used the blood brain DNA methylation comparison tool to analyze the methylation of Dlg4. The results obtained using the cg03613822 probe indicated that methylation was present (P < 0.01) in four regions, including the frontal cortex (PFC), entorhinal cortex (EC), superior temporal gyrus (STG), and cerebellum (CER), and that the degree of methylation in the cerebellum was lower than that in three other regions (Fig. 6B). This result suggests that methylation of the Dlg4 gene may be involved in the neuroprotective effect mediated by FNS.

Although specific DNA sequences that affect Dlg4 methylation have not been described, we utilized the PhenoGen database, a rat brain-wide gene expression database that analyzes the correlations between a phenotype and a central nervous system function and uses behavioral and expression QTL data to identify the core genes. This approach was employed to identify the chromosomal region corresponding to the location of the expression trait locus. The results indicated that Dlg4 is mainly located on chromosome 10 in the rat and that there is a significant correlation between the DNA sequences of chromosomes 3, 5, 11, 15, and 20 and the X chromosome of the rat and the Dlg4 gene (Fig. 6C). Thus, hypomethylation of Dlg4 in the cerebellum may be influenced by certain sequences on these chromosomes, which could potentially upregulate relevant neuroprotective genes.

### Analysis of the Core Gene by the Gene Ontology Analysis and ROC Curves

**Fig. 7:**
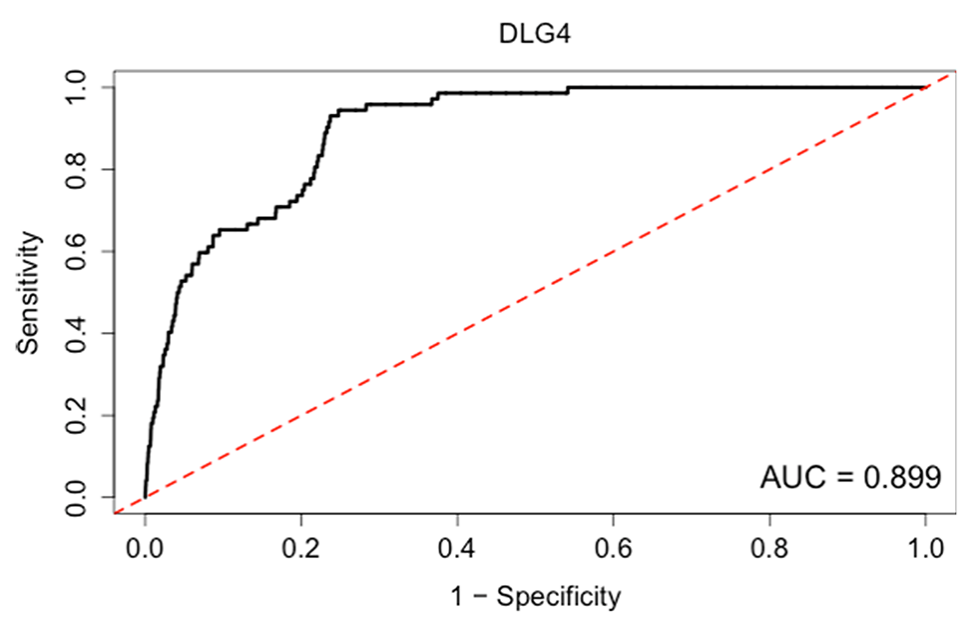
Functional annotation and credibility analysis of Dlg4

Analysis of the core Dlg4 gene indicated that the functions of Dlg4 include short-term regulation of synaptic plasticity, maturation of synaptic vesicles, and positive regulation of calcium-dependent exocytosis, which are mainly involved in the regulation of synaptic vesicles released by neurotransmitters (Table 2). Additionally, the Dlg4 gene is involved in synaptic plasticity related to NMDA receptor signal transduction. Experimental studies demonstrated that overexpression or depletion of Dlg4 can change the ratio of excitatory synapses to inhibitory synapses in hippocampal neurons [44]. The area under the ROC curve was 0.899 suggesting good sensitivity and specificity and indicating that it is highly likely that Dlg4 regulates the function of synaptic vesicles in the nervous system (Fig. 7).

**Table 2:** Gene Ontology analysis of Dlg4

GO term	Gene Set	Z-score
GO:0048172	regulation of short-term neuronal synaptic plasticity	6.70037
GO:0016188	synaptic vesicle maturation	5.982374
GO:0045956	positive regulation of calcium ion-dependent exocytosis	5.947627
GO:2000463	positive regulation of excitatory postsynaptic membrane potential	5.793748
GO:0016081	synaptic vesicle docking involved in exocytosis	5.669579
GO:0035641	locomotory exploration behavior	5.556733
GO:2000311	regulation of AMPA selective glutamate receptor act	5.513886
GO:0014047	glutamate secretion	5.421376
GO:0016079	synaptic vesicle exocytosis	5.417028
GO:2000300	regulation of synaptic vesicle exocytosis	5.272977

### APA analysis

The untranslated regions (UTRs) of mRNAs are critical sites for regulatory control, as they can affect mRNA stability, localization and translation. To gain a better understanding of transcriptional regulation, we explore how alternative cleavage and polyadenylation alter the fate of mRNAs by altering the length of their 3’UTRs [45]. We began to evaluate non-coding regions of genes thanks in part to the discovery of miRNAs and the importance of miRNA-mRNA interactions in gene regulation. Genes carry multiple polyadenylation signals in their 3’-UTR (untranslated region), which can be differentially selected depending on the physiological state of the cell, resulting in alternative 3’-UTR isoforms. Consequently, we examine the mechanism of APA events after stroke by linking it to disease genes [37].

**Fig. 8:**
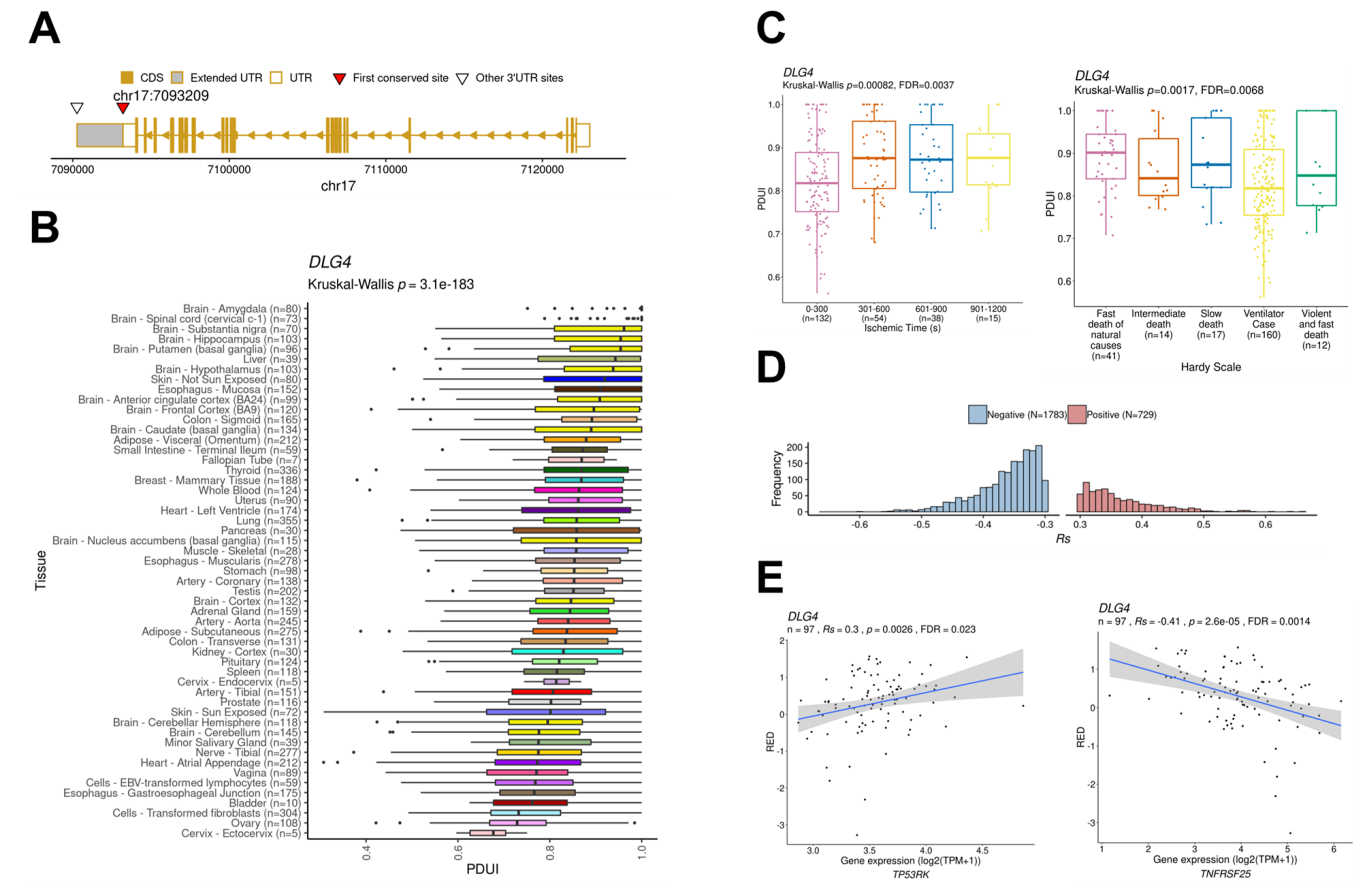
APA event analysis of Dlg4. **A** Mapping of the APA locus of the Dlg4 gene. **B** APA event map of the Dlg4 gene in various brain tissues. **C** Ischemic PDUI and death degree of Dlg4 gene Hardy score in different periods. **D** Distribution of positive and negative correlation genes of Dlg4 gene PDUI-related gene expression. **E** The PDUI of Dlg4 was positively correlated with the expression of the anti-apoptotic gene TP53RK, and negatively correlated with the expression of the apoptosis factor TNFRSF25. The percentage of distal poly-A site usage index (PDUI) was defined as the proportion of transcripts with distal poly-A sites (used to quantify APA events). Significant correlations between gene expression levels and APA use were defined as |Rs| > 0.3 and |FDR| < 0.05

We quantified APA using the DaPars algorithm and found that the APA site of the Dlg4 gene was located at chr17:7093469-7093932 in the first exon of the 3’-UTR (Fig. 8A). Meanwhile, in each tissue, the Percentage of Distal poly A site Usage Index (PDUI) of Dlg4 gene was significantly different between brain and other tissues, and brain tissues were more prone to ARA events than other tissues (Fig. 8B). In whole blood, the probability of APA events in Dlg4 gene in different tissues varied with different periods of ischemia (Fig. 8C). The probability of APA events in Dlg4 was lower in ischemia 0-300 s than in later periods. After the early ischemic stimulus, the occurrence of APA events stabilized within 300–1200 s, indicating the presence of distinct APA events in vivo at the beginning. In the death category of the Hardy scale, the probability of APA events occurring during hypoxia is the highest, which is higher than that of rapid natural death and chronic death. Therefore, we evaluated that the Dlg4 gene is prone to trigger APA events during ischemia and hypoxia. The histogram shows the number distribution of positive and negative correlations with Dlg4 gene expression, and the number of negatively correlated genes is more than that of positively correlated genes (Fig. 8D). The expression of factor TNFRSF25 was negatively correlated (Fig. 8E), indicating that the APA event of Dlg4 also plays a positive role in anti-apoptosis during ischemia and hypoxia.

## Discussion

Recent research has demonstrated the neuroprotective effects of FNS and suggested that FNS preconditioning can significantly enhance resistance to ischemic brain injury. miRNAs are the general regulators of gene expression at the posttranslational level, and many miRNAs respond to preconditioning stimuli [46, 47]. Brain pre-stimulation is known to regulate miRNA expression, leading to loss of synaptic inhibitory nerve transmission [16]. Therefore, it is possible that miRNAs play a role in the FNS-induced neuroprotective cascade, which reduces the extent of reduces ischemic brain damage.

To explore whether continuous time-series data can provide a better understanding of complex regulatory interactions, we collected a dataset of time-series miRNAs representing changes in electrical stimulation, and analyzed the data sets at different time points after transcriptional activation. We identified miRNAs differentially regulated by FNS and analyzed the neural circuits activated by electrical stimulation of the parietal nucleus, revealing how it confers neuroprotection by modulating neurotransmitter release. Our investigation also identified UBTF as the predominant transcription factor, while MAPK1 was determined to be the most pertinent kinase involved in this process due to its ability to regulate apoptosis. Network topology analysis identified Dlg4, which regulates synaptic vesicles, as the central gene and determined that chromosomes 3, 5, 11, 15, and 20 and the X chromosome of rat may have a profound influence on hypomethylation of Dlg4 in the cerebellum.

The Dlg4 gene encodes postsynaptic density protein 95 (PSD95), a major synaptic protein that aggregates glutamate receptors and plays a key role in regulating glutamate receptor density and activity because it acts as the backbone of protein complexes that mediate signaling between membrane glutamate receptors and intracellular pathways [48–51]. Therefore, the effect of FNS may be mediated by various cytokines and intercellular signaling networks, and FNS stimulates the inherent self-protective ability of the brain to reduce the injury [52].

Transcription factors and kinases play a crucial role in various upstream regulatory pathways, affecting differentiation, proliferation, and apoptosis [53]. UBTF transcription factor and MAPK1 kinase are associated with the target genes. UBTF is a key gene related to neurodegenerative diseases and brain atrophy. Abnormal expression of UBTF can cause an increase in the DNA double-strand breaks in fibroblasts and an increase in apoptotic cell death [54]. In cultured rat neurons, phosphorylation of MAPK1 can influence the duration of MAPK1 activation and downstream signal transduction [55]. Downregulation of MAPK1 can promote the recovery of motor coordination and integration after cerebral ischemia injury in rats [56]. Combination of the results of complete upstream pathway analyses suggests that transcription factors and protein kinases, such as UBTF and MAPK1, may be involved in the reduction of cerebral infarction volume after FNS. This discovery deepens our understanding of the potential upstream molecular mechanisms underlying the effects of FNS at the transcriptional level.

The present study’s data suggest that FNS significantly affects gene expression across multiple neuroprotective pathways in various brain regions, including those involved in neurotransmission such as glutamate, serotonin, and dopamine neurotransmitter pathways. Notably, the distribution of several neurotransmitter cycle pathways was highly coincident, and this effect may be related to the metabolic release of neurotransmitters in the neuronal system induced by FNS. The information obtained by integrating time series miRNA data may represent an important step in understanding the principle of FNS regulation. Our findings highlight a unique pattern of miRNA-associated pathways involved in FNS-induced protection against brain injury, underscoring their potential clinical applicability.

**Fig. 9:**
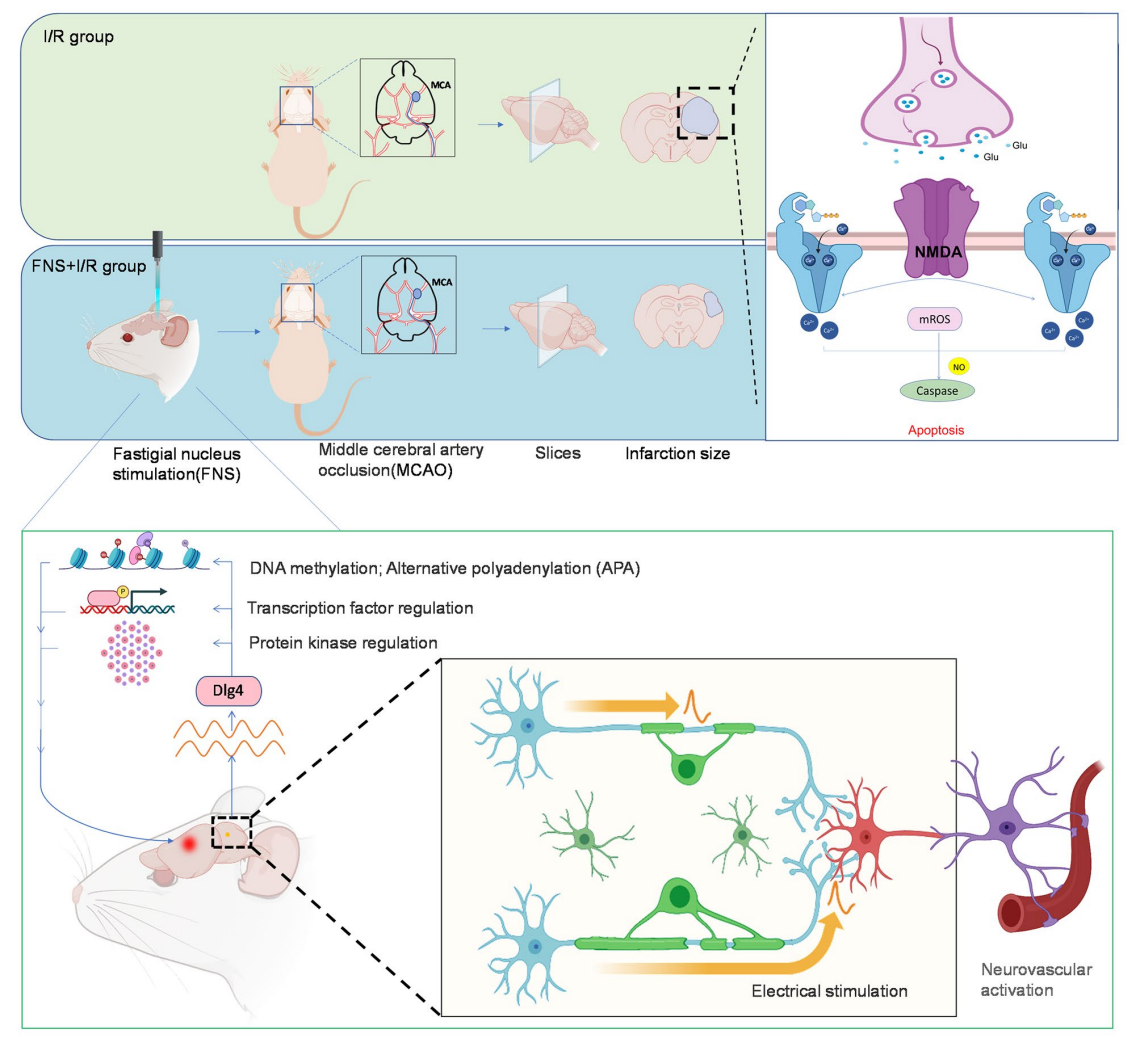
Hypothesized mechanistic diagram of neuroprotection from cerebellar fastigial nucleus

Both excitatory neurotransmitter glutamate and monoamines, such as dopamine and serotonin, have been shown to regulate neuronal excitability and affect the concentration of glutamate in brain regions like the cortex and hypothalamus. Moreover, neurotransmitters can protect marginally damaged neurons by reducing neuronal activity and ion flow or increasing local metabolism around the injury [57]. Therefore, we suggest that this endogenous neuroprotective mechanism is a result of neurotransmitter release. FNS may promote neuroprotection by decreasing local metabolism and neural excitability through reducing the release of neurotransmitters, including glutamate (Fig. 9) [5, 58–61].

DNA methylation of specific genomic sites in the brain has a significant association with DNA methylation levels in blood samples [62]. Notably, using the cg03613822 probe, we observed that Dlg4 methylation in the cerebellum exhibited distinct differences compared to other brain regions, and individual differences in the whole blood were significantly correlated with differences detected in all four tested brain regions. Methylated genes regulate the expression of the genes related to neuroprotection by changing the structure of the chromatin and promoting the binding of the transcription factors [63, 64]. Furthermore, our analysis revealed that Dlg4 is situated on rat chromosome 10 of the rat and that DNA sequences on chromosomes 3, 5, 11, 15, and 20 and the X chromosome participate in the neuroprotective effect of FNS by regulating Dlg4 expression. These findings suggest that hypomethylation of the core gene Dlg4 in the cerebellum, along with further investigation into miRNA regulation, could offer valuable insights into the underlying mechanisms involved in FNS and deepen our understanding of the inherent protective abilities of the brain.

Alternative polyadenylation (APA) has a significant impact on the transformation of biological processes, with many biological processes, such as B cell activation, tumorigenesis, and proliferation, intricately linked to global APA events in the mRNA 3’ UTR. Advances in sequencing and transcriptome analysis techniques have paved the way for groundbreaking discoveries that continue to shed light on disease complexity. Transcription plays a critical role in shaping the functional diversity of cells [65].

Analysis of Dlg4 revealed that brain tissue had the highest frequency of APA events. The likelihood of APA events gradually increased during the early period of ischemia and then stabilized. Furthermore, the probability of APA events was greater under hypoxic conditions, prompting us to investigate the susceptibility of Dlg4 gene to trigger APA events during ischemia and hypoxia. When we correlated gene expression, we discovered that Dlg4 exhibited a positive correlation with the expression of anti-apoptotic genes, but negatively correlated with the expression of apoptotic factors, indicating that Dlg4 played a protective role in regulating the body’s stress response during APA events.

The present study has some limitations that should be acknowledged. These include the model’s limited clinical relevance to human stroke patients, possible variability of results due to differences in surgical techniques and anesthesia, unclear mechanisms by which Dlg4 provides neuroprotection, and a lack of evaluation of potential effects beyond the brain. Our study may have the following limitations:

(1) Specificity of the model: Although the model aims to simulate ischemic brain injury, it is still an artificial manipulation that does not exactly mimic human stroke situations. (2) Reproducibility of results: Variations in surgical techniques, duration, frequency, or intensity of electrical stimulation, and anesthesia could lead to inconsistent outcomes across different experiments from various labs, challenging the reproducibility of any observed neuroprotective effect or associating benefits with Dlg4 [66]. (3) Attribute of findings problem: Although the present study suggests the role of Dlg4 in neuroprotection after ischemia/reperfusion induced by electrical stimulation of the fastigial nucleus, the actual mechanism by which Dlg4 provides neuroprotection is unclear.

Further research is necessary to address these limitations and refine the model. One avenue of research could involve exploring alternative models for simulating ischemia/reperfusion injury with greater clinical relevance to human stroke situations. Moreover, studies focused on elucidating the underlying molecular mechanisms by which Dlg4 confers neuroprotection may help refine approaches for stimulating the fastigial nucleus. Conducting larger animal studies with greater anatomical similarity to humans, such as primate models [67], can also increase translatability to clinical practice. Additionally, further research could explore other neuroprotective interventions and therapies for post-stroke recovery and rehabilitation, including pharmacological treatments and non-invasive neuromodulation techniques like transcranial magnetic stimulation (TMS) [68]. In summary, while the present study suggests some promise for using electrical stimulation of the fastigial nucleus to provide neuroprotection in ischemia-reperfusion injury in rat, further studies are required to improve the clinical relevance and optimize methods for safe and effective application in stroke patients [69].

In the nervous system, Dlg4 regulates the release probability of synaptic transmitter vesicles, alters short-term presynaptic plasticity [70], and enhances postsynaptic aggregation and glutamate receptor activity [71]. Therefore, in ischemic brain injury, FNS can activate neuronal cells in the hypothalamus and other brain regions, and regulate the release of neurotransmitters by targeting Dlg4 with miRNAs, and at the same time induce APA events to play an anti-apoptotic role, thereby regulating neural synapses in the brain and realizing neuroprotection. In our future research, utilizing in situ hybridization (ISH) to detect the expression levels of miRNA and core genes in surviving cortical neurons will address some of the inherent limitations of relying primarily on high-throughput sequencing and bioinformatics analysis. If the histological findings are consistent with our current research results, it will convincingly demonstrate the regulatory role of FNS on ischemic cortical neuron gene expression mediated by miRNA, which holds important value for translating our research into clinical applications.

## Conclusion

This study provides the first description of the effect of FNS on miRNA expression profile after cerebral ischemia/reperfusion, including timepoints of 3, 6, 12, 24, and 72 h. Furthermore, the study expands on the enrichment analysis of upstream transcription factors and kinases, explores hypomethylation and regulation of APA events of the core gene Dlg4 (PSD95) in the cerebellum, and investigates its effect on the downstream pathway in neurotransmitter release. Our study reveals that FNS may activate neuronal and neurovascular coupling by regulating the release of neurotransmitters in synaptic vesicles through the methylation of core Dlg4 gene and the corresponding transcription factors and protein kinases, inducing the anti-apoptotic mechanism of APA events, thereby protecting neurons from ischemic injury.

## Electronic supplementary material

Below is the link to the electronic supplementary material.

Supplementary Material 1: Table S1: Differentially expressed miRNA in fastigial nucleus stimulation (FNS) group at 3 h, 6 h, 12 h, 24 h and 72 h

## Data Availability

The datasets generated and analyzed during the current study are available in the figshare repository, 10.6084/m9.figshare.22461610.v1.
