# Supplementary material for: Transcriptome analysis reveals the neuroprotective effect of Dlg4 against fastigial nucleus stimulation-induced ischemia/reperfusion injury in rats
Source: BMC Neurosci. 2023 Jul 31;24:40. doi: 10.1186/s12868-023-00811-6 (PMC10391810; doi:10.1186/s12868-023-00811-6)
Supplement: Supplementary file 1 — Supplementary Material 1: Table S1: Differentially expressed miRNA in fastigial nucleus stimulation (FNS) group at 3 h, 6 h, 12 h, 24 h and 72 h [file 12868_2023_811_MOESM1_ESM.docx]

Supplementary material

for

**Transcriptome Analysis Reveals the Neuroprotective Effect of Dlg4 Against Fastigial Nucleus Stimulation-Induced Ischemia/Reperfusion Injury in Rats**

**Table S1:** Differentially expressed miRNA in fastigial nucleus stimulation (FNS) group at 3 h, 6 h, 12 h, 24 h and 72 h

The rats were randomly divided into two groups: (1) the I/R control group (right middle cerebral artery occlusion for 2 h followed by reperfusion for 3, 6, 12, 24, and 72 h; 5 rats at each time point) and (2) I/R+FNS group (electrical stimulation of the right cerebellar fastigial nucleus for 1 h, subsequent cerebral ischemia for 2 h, and reperfusion for 3, 6, 12, 24, and 72 h; 5 rats at each time point),then, pairwise comparison between the groups (I/R control-VS- I/R+FNS group) was used to identify differentially expressed miRNAs (DEMs) using DESeq (P < 0.05 and |log2fold change| > 1)

| 3h |  |  | 6h |  |  | 12h |  |  | 24h |  |  | 72h |  |  |
| --- | --- | --- | --- | --- | --- | --- | --- | --- | --- | --- | --- | --- | --- | --- |
| Reporter Name | p-value | Log2 (G2/G1) | Reporter Name | p-value | Log2 (G2/G1) | Reporter Name | p-value | Log2 (G2/G1) | Reporter Name | p-value | Log2 (G2/G1) | Reporter Name | p-value | Log2 (G2/G1) |
| rno-miR-3574 | 2.91E-05 | 1.833954 | rno-miR-384-5p | 3.68E-06 | 1.168961 | rno-miR-758 | 2E-05 | 1.963696 | rno-miR-26b | 3.05E-06 | -1.05978 | rno-miR-1224 | 7.19E-06 | 1.481148 |
| rno-miR-181c | 5.71E-05 | 1.445143 | rno-miR-672 | 4.25E-05 | 2.300882 | rno-miR-137 | 8.37E-05 | -1.81419 | rno-miR-34a | 2.39E-05 | 0.611878 | rno-miR-466b | 2.35E-05 | 3.502375 |
| rno-miR-328a | 7.23E-05 | -1.38361 | rno-miR-539 | 4.85E-05 | 0.913495 | rno-miR-99a | 8.63E-05 | -0.94026 | rno-miR-466b | 3.09E-05 | 2.493203 | rno-miR-210 | 2.79E-05 | 1.281053 |
| rno-miR-365 | 0.000107 | 2.021163 | rno-miR-451 | 8.46E-05 | -2.09875 | rno-miR-29c | 0.000117 | -3.00186 | rno-miR-335 | 6.19E-05 | -0.97308 | rno-miR-3584-5p | 6.7E-05 | 1.850101 |
| rno-miR-335 | 0.000127 | 1.451186 | rno-miR-466b | 8.68E-05 | -1.58361 | rno-miR-3574 | 0.000121 | 2.338733 | rno-miR-320 | 6.71E-05 | 2.829823 | rno-miR-483 | 8.43E-05 | 1.457258 |
| rno-miR-31 | 0.00017 | -1.0375 | rno-miR-425 | 9.47E-05 | -1.2993 | rno-miR-218a | 0.000154 | -1.53008 | rno-miR-423 | 9.09E-05 | 1.33808 | rno-miR-32 | 0.00012 | 2.915421 |
| rno-miR-16 | 0.000192 | -0.92954 | rno-miR-98 | 0.000108 | 1.822885 | rno-miR-22 | 0.000185 | -0.58611 | rno-miR-568 | 0.00013 | 0.447861 | rno-miR-296 | 0.00012 | 2.083524 |
| rno-miR-758 | 0.000195 | 1.689728 | rno-miR-598-3p | 0.000133 | 0.627298 | rno-miR-487b | 0.000193 | 0.521131 | rno-miR-21 | 0.000153 | -1.45641 | rno-miR-466b-1 | 0.000197 | 0.755763 |
| rno-miR-208 | 0.000236 | 1.540082 | rno-miR-26b | 0.000303 | 1.643358 | rno-miR-30a | 0.000232 | -0.72399 | rno-miR-30b-5p | 0.000155 | -0.83295 | rno-miR-365 | 0.000201 | 1.411142 |
| rno-miR-182 | 0.000286 | 1.42203 | rno-miR-16 | 0.00044 | -0.47998 | rno-miR-466b | 0.000251 | -1.33527 | rno-miR-3574 | 0.000193 | 0.827072 | rno-miR-34c | 0.000216 | 1.226495 |
| rno-miR-146b | 0.000342 | 0.881685 | rno-miR-143 | 0.000698 | -0.85321 | rno-miR-1224 | 0.000281 | -1.59852 | rno-miR-218a | 0.000234 | -0.61061 | rno-miR-187 | 0.000222 | -1.45563 |
| rno-miR-212 | 0.000355 | -0.68088 | rno-miR-212 | 0.000713 | -0.95507 | rno-miR-139-5p | 0.000339 | 1.109976 | rno-miR-92b | 0.000248 | 0.905443 | rno-miR-214 | 0.000224 | 2.089817 |
| rno-miR-210 | 0.000361 | 1.88669 | rno-miR-139-5p | 0.000851 | 0.489719 | rno-miR-365 | 0.000361 | 1.027914 | rno-miR-758 | 0.000248 | 1.071033 | rno-miR-494 | 0.000249 | 3.378372 |
| rno-miR-320 | 0.000362 | -0.23059 | rno-let-7d | 0.000876 | 0.876606 | rno-miR-196c | 0.000441 | 1.536589 | rno-miR-101a | 0.000252 | -0.96452 | rno-miR-466c | 0.000471 | 0.717258 |
| rno-miR-539 | 0.000405 | 0.395968 | rno-miR-181d | 0.000948 | 0.905809 | rno-miR-129-1 | 0.000491 | -2.52915 | rno-miR-129-1 | 0.000314 | -1.29199 | rno-miR-466b-2 | 0.000505 | 0.743444 |
| rno-miR-99a | 0.000494 | -0.74007 | rno-miR-7a | 0.001128 | 1.085225 | rno-miR-210 | 0.000556 | 1.445908 | rno-miR-126 | 0.000325 | -0.52855 | rno-miR-188 | 0.000509 | 1.314929 |
| rno-miR-99b | 0.000526 | -0.61757 | rno-miR-29a | 0.001146 | -0.81105 | rno-miR-29a | 0.00058 | -0.72832 | rno-miR-196c | 0.000364 | 1.382754 | rno-miR-3574 | 0.000655 | 0.971112 |
| rno-miR-93 | 0.000568 | -1.1892 | rno-miR-106b | 0.001427 | -0.7497 | rno-miR-672 | 0.000696 | -1.11571 | rno-miR-129-2 | 0.000442 | -0.7823 | rno-miR-21 | 0.000691 | -0.70194 |
| rno-miR-1224 | 0.000593 | 1.001678 | rno-miR-346 | 0.001486 | -0.37057 | rno-miR-290 | 0.000815 | 0.572805 | rno-miR-494 | 0.000464 | -0.7153 | rno-miR-221 | 0.000728 | -0.56291 |
| rno-miR-352 | 0.000622 | 0.972957 | rno-miR-29c | 0.00151 | -1.01888 | rno-miR-543 | 0.000829 | -1.35329 | rno-miR-382 | 0.000534 | 1.416663 | rno-miR-758 | 0.000746 | 1.046079 |
| rno-miR-30d | 0.000652 | -0.73922 | rno-miR-352 | 0.001665 | 1.639651 | rno-miR-181a | 0.000861 | -0.44839 | rno-miR-214 | 0.000719 | 0.819404 | rno-miR-150 | 0.00099 | -0.82642 |
| rno-miR-494 | 0.000698 | 2.034055 | rno-miR-328a | 0.001687 | -1.23743 | rno-miR-98 | 0.00095 | -1.12615 | rno-miR-30d | 0.000739 | -0.67589 | rno-miR-143 | 0.001023 | 0.325347 |
| rno-miR-330 | 0.000771 | -0.94303 | rno-miR-30e | 0.002105 | -0.27056 | rno-miR-9 | 0.000961 | -0.90864 | rno-miR-328a | 0.000765 | 1.140115 | rno-miR-543 | 0.001233 | -1.09279 |
| rno-miR-338 | 0.000773 | 0.85047 | rno-miR-22 | 0.002246 | -0.49362 | rno-miR-30b-5p | 0.001056 | -1.05016 | rno-miR-98 | 0.000773 | -1.21515 | rno-miR-328a | 0.001679 | 1.948296 |
| rno-miR-34a | 0.000781 | -0.89741 | rno-miR-376b-3p | 0.002647 | 0.727768 | rno-miR-320 | 0.001318 | 0.602794 | rno-miR-320 | 0.000781 | 0.677777 | rno-miR-145 | 0.001728 | 0.399285 |
| rno-miR-342-3p | 0.000794 | -0.41631 | rno-miR-34a | 0.002713 | -0.76902 | rno-miR-214 | 0.00172 | 0.886509 | rno-miR-409-3p | 0.000808 | 0.334936 | rno-miR-346 | 0.002266 | 1.332352 |
| rno-miR-129-1 | 0.000906 | -0.98134 | rno-miR-129-1 | 0.002821 | -1.38706 | rno-miR-652 | 0.002005 | -0.81507 | rno-miR-346 | 0.000818 | 1.34065 | rno-miR-139-5p | 0.002463 | -0.89012 |
| rno-miR-137 | 0.000975 | -0.77981 | rno-miR-331 | 0.002875 | -0.37065 | rno-miR-7a | 0.002059 | -0.87969 | rno-miR-181b | 0.000828 | -0.30095 | rno-miR-146b | 0.002547 | -0.44248 |
| rno-miR-181d | 0.000976 | 1.676843 | rno-miR-93 | 0.002956 | -0.42395 | rno-miR-328a | 0.002075 | -0.49571 | rno-miR-410 | 0.000979 | -0.70332 | rno-miR-30b-5p | 0.003111 | -0.58923 |
| rno-miR-187 | 0.000991 | 1.549307 | rno-miR-3584-5p | 0.003009 | -0.2092 | rno-miR-16 | 0.002215 | -0.44097 | rno-let-7e | 0.001137 | -0.44771 | rno-miR-9 | 0.003215 | -0.39124 |
| rno-miR-410 | 0.001075 | -1.02009 | rno-miR-674-5p | 0.00308 | -0.3255 | rno-miR-27b | 0.002391 | 0.316646 | rno-miR-3591 | 0.001474 | 2.120023 | rno-miR-30d | 0.003533 | -0.47608 |
| rno-miR-26b | 0.001079 | 1.251021 | rno-miR-30d | 0.003867 | -0.5486 | rno-miR-140 | 0.002461 | 0.40767 | rno-miR-539 | 0.001491 | -0.29042 | rno-miR-423 | 0.003615 | 1.848454 |
| rno-miR-331 | 0.0011 | -0.42067 | rno-miR-25 | 0.004641 | -0.34989 | rno-miR-129-2 | 0.002547 | -1.22953 | rno-miR-137 | 0.001523 | -0.73978 | rno-miR-29b | 0.003769 | 0.220225 |
| rno-miR-568 | 0.001136 | 1.34999 | rno-let-7e | 0.004659 | 0.711401 | rno-miR-208 | 0.002598 | 1.022977 | rno-miR-365 | 0.001567 | -1.93652 | rno-miR-218a | 0.004279 | -0.81086 |
| rno-miR-196c | 0.001267 | 1.414291 | rno-miR-335 | 0.005383 | 1.153349 | rno-miR-346 | 0.002878 | 0.672857 | rno-miR-210 | 0.001714 | 1.068119 | rno-miR-30c | 0.004493 | -0.45422 |
| rno-miR-92b | 0.001383 | -0.84016 | rno-miR-328a | 0.005502 | -1.93002 | rno-miR-23b | 0.002916 | 0.636186 | rno-miR-185 | 0.001796 | 0.404856 | rno-miR-485 | 0.004596 | 0.704342 |
| rno-miR-145 | 0.001551 | -0.62307 | rno-miR-187 | 0.005685 | -0.58713 | rno-miR-30d | 0.002969 | -0.63199 | rno-miR-181d | 0.001827 | -0.47858 | rno-miR-3564 | 0.004655 | 1.540638 |
| rno-miR-100 | 0.001558 | -0.29048 | rno-miR-758 | 0.00582 | 1.297038 | rno-miR-337 | 0.003008 | 0.444054 | rno-miR-16 | 0.001838 | -0.27579 | rno-miR-9 | 0.004899 | -0.35414 |
| rno-miR-324-5p | 0.001609 | -0.58755 | rno-miR-151 | 0.006045 | -0.38533 | rno-miR-129 | 0.003203 | -0.73765 | rno-miR-122 | 0.001885 | 2.558323 | rno-miR-327 | 0.004938 | 1.727483 |
| rno-miR-344a-3p | 0.001902 | -1.21508 | rno-miR-219-2-3p | 0.006446 | 0.446013 | rno-miR-34c | 0.003243 | 1.081762 | rno-miR-384-5p | 0.001945 | -0.29593 | rno-miR-598-3p | 0.005257 | -0.45948 |
| rno-miR-328a | 0.001956 | 3.384511 | rno-miR-146b | 0.007169 | 0.354571 | rno-miR-29b | 0.003373 | -2.99748 | rno-miR-376a | 0.002058 | 1.12265 | rno-miR-495 | 0.00535 | -0.58005 |
| rno-miR-32 | 0.002203 | 0.846722 | rno-miR-361 | 0.007922 | 0.384478 | rno-miR-32 | 0.003478 | 0.836506 | rno-miR-29c | 0.002215 | -1.07859 | rno-miR-20a | 0.007339 | -0.35615 |
| rno-miR-126 | 0.002294 | -0.33714 | rno-miR-383 | 0.007954 | -0.42438 | rno-let-7e | 0.003801 | 0.325013 | rno-miR-195 | 0.002441 | -0.59765 | rno-miR-125a-5p | 0.007549 | -0.6364 |
| rno-miR-325-3p | 0.002343 | -0.75875 | rno-miR-31 | 0.008008 | -0.32625 | rno-miR-30c | 0.004028 | -0.43415 | rno-miR-344a-3p | 0.002747 | 0.694189 | rno-miR-187 | 0.008163 | 0.857922 |
| rno-miR-24 | 0.002483 | -0.41445 | rno-miR-29b | 0.008097 | -0.87884 | rno-miR-23a | 0.004141 | 0.833333 | rno-miR-323 | 0.003044 | -0.81387 | rno-miR-222 | 0.008187 | -0.60611 |
| rno-miR-324-3p | 0.002529 | -0.77043 | rno-miR-138 | 0.008287 | -0.45623 | rno-miR-324-3p | 0.004162 | 0.325139 | rno-miR-485 | 0.003499 | 1.094023 | rno-miR-384-5p | 0.009913 | -0.73665 |
| rno-miR-21 | 0.002689 | 1.836158 | rno-let-7i | 0.009048 | 0.424641 | rno-miR-494 | 0.004571 | -1.11755 | rno-miR-352 | 0.00351 | -0.39227 | rno-miR-199a-3p | 0.000298 | -0.97426 |
| rno-let-7d | 0.00272 | 0.862777 | rno-miR-181a | 0.009736 | -0.41767 | rno-miR-125b-5p | 0.004941 | -0.6533 | rno-miR-384-3p | 0.003675 | -0.93851 | rno-miR-215 | 0.000425 | 1.441297 |
| rno-miR-487b | 0.003032 | -0.42458 | rno-miR-329 | 8.98E-05 | -0.90681 | rno-miR-378 | 0.005011 | 0.496881 | rno-miR-146b | 0.003873 | -0.31937 | rno-miR-380 | 0.000774 | -1.14298 |
| rno-miR-674-3p | 0.003154 | -1.35613 | rno-miR-3588 | 0.000149 | 3.178269 | rno-miR-126 | 0.005328 | -0.46997 | rno-miR-29b | 0.003945 | -0.48028 | rno-miR-199a-5p | 0.001007 | -10.6878 |
| rno-miR-384-3p | 0.003329 | 0.925806 | rno-miR-219-5p | 0.000217 | 9.83267 | rno-miR-324-5p | 0.005337 | 0.522193 | rno-miR-379 | 0.00427 | 0.129252 | rno-miR-101a | 0.001042 | -0.89615 |
| rno-miR-154 | 0.003377 | 0.524317 | rno-miR-215 | 0.000244 | 1.564726 | rno-miR-3564 | 0.005357 | 1.000891 | rno-miR-138-1 | 0.004304 | 0.649436 | rno-miR-665 | 0.001134 | 1.86067 |
| rno-miR-7a | 0.003496 | 0.63227 | rno-miR-665 | 0.000358 | -0.85768 | rno-miR-125a-5p | 0.006007 | -0.51374 | rno-miR-32 | 0.004348 | 2.078626 | rno-miR-499 | 0.001413 | -10.4979 |
| rno-miR-425 | 0.00447 | -1.5325 | rno-miR-125a-3p | 0.000416 | 0.577272 | rno-let-7d | 0.006709 | 0.30276 | rno-miR-30e | 0.004455 | -0.57966 | rno-miR-1 | 0.001423 | 1.386491 |
| rno-miR-219-2-3p | 0.004666 | 0.657393 | rno-miR-935 | 0.000526 | 9.447239 | rno-miR-331 | 0.007171 | -0.48455 | rno-miR-324-5p | 0.004504 | 0.392937 | rno-miR-18a | 0.001463 | -11.2556 |
| rno-miR-195 | 0.004726 | 0.447904 | rno-miR-376b-5p | 0.000738 | 0.537099 | rno-miR-568 | 0.007356 | 1.672014 | rno-miR-338 | 0.005113 | -0.37108 | rno-miR-211 | 0.001653 | 1.690826 |
| rno-miR-29a | 0.005078 | -0.37917 | rno-miR-200c | 0.00076 | -2.59091 | rno-miR-150 | 0.007539 | -0.43564 | rno-miR-330 | 0.005231 | 0.591876 | rno-miR-543 | 0.001777 | -1.87935 |
| rno-miR-138 | 0.005612 | -0.55296 | rno-miR-153 | 0.00123 | -1.05692 | rno-miR-27a | 0.007971 | 0.476574 | rno-miR-182 | 0.005355 | 1.065963 | rno-miR-92a | 0.002013 | -0.95466 |
| rno-miR-379 | 0.005623 | -0.1913 | rno-miR-345-5p | 0.001318 | -0.71286 | rno-miR-24 | 0.008039 | 0.317566 | rno-miR-376b-3p | 0.005868 | -0.61855 | rno-miR-139-3p | 0.002211 | -0.63315 |
| rno-miR-204 | 0.005788 | 1.258991 | rno-miR-369-5p | 0.001359 | 0.691857 | rno-miR-384-5p | 0.008082 | 0.672892 | rno-miR-342-3p | 0.00596 | -0.41521 | rno-miR-1249 | 0.002369 | 1.769904 |
| rno-miR-143 | 0.006531 | -0.66439 | rno-miR-541 | 0.001481 | -11.4861 | rno-miR-128 | 0.008219 | -0.44093 | rno-miR-150 | 0.006062 | -0.47146 | rno-miR-101b | 0.002521 | -0.93206 |
| rno-miR-3584-5p | 0.007243 | 1.700645 | rno-miR-504 | 0.001678 | -0.87497 | rno-miR-425 | 0.00895 | -1.08043 | rno-miR-383 | 0.00641 | 0.394184 | rno-miR-411 | 0.002569 | -1.19501 |
| rno-miR-98 | 0.007755 | 1.723471 | rno-miR-411 | 0.002048 | -0.96117 | rno-let-7b | 0.009201 | 0.403654 | rno-miR-466b-1 | 0.0069 | 0.614662 | rno-miR-204 | 0.002669 | 1.367641 |
| rno-miR-214 | 0.008018 | 0.841476 | rno-miR-369-3p | 0.002204 | 2.49969 | rno-miR-151 | 0.009815 | 0.422591 | rno-miR-466b-2 | 0.0073 | 0.685401 | rno-miR-664-1 | 0.002688 | 0.576645 |
| rno-miR-140 | 0.008326 | -0.31491 | rno-miR-298 | 0.002449 | 1.391804 | rno-miR-218a-2 | 6.94E-05 | -12.6888 | rno-miR-3564 | 0.007526 | 0.406522 | rno-miR-3541 | 0.003395 | 1.918945 |
| rno-miR-384-5p | 0.00864 | 0.712348 | rno-miR-431 | 0.002727 | -0.77915 | rno-miR-3558-3p | 0.000272 | 11.92422 | rno-miR-483 | 0.007654 | 0.700286 | rno-miR-3552 | 0.003548 | 0.998265 |
| rno-miR-483 | 0.009766 | 0.432666 | rno-miR-497 | 0.002929 | -1.05826 | rno-miR-219-1-3p | 0.000317 | -12.5114 | rno-miR-212 | 0.008414 | -0.47942 | rno-miR-3573-3p | 0.004083 | 1.943684 |
| rno-miR-153 | 0.000139 | -1.13907 | rno-miR-434 | 0.003034 | -0.7812 | rno-miR-675 | 0.000321 | -13.6108 | rno-miR-138 | 0.009249 | 0.30139 | rno-miR-871 | 0.004179 | -10.7272 |
| rno-miR-770 | 0.00035 | -0.74292 | rno-miR-484 | 0.003121 | 1.209868 | rno-miR-137 | 0.000357 | 0.946275 | rno-miR-543 | 0.009935 | -0.34087 | rno-miR-181a-2 | 0.00432 | 2.159648 |
| rno-miR-7a-1 | 0.000517 | -0.93069 | rno-miR-17-5p | 0.003475 | -0.37788 | rno-let-7a-1 | 0.000953 | 2.357695 | rno-miR-196b | 3.91E-05 | 1.74949 | rno-miR-138-2 | 0.005796 | -0.49294 |
| rno-miR-206 | 0.000756 | 1.653792 | rno-miR-344b-5p | 0.003534 | -0.9284 | rno-let-7f-1 | 0.001908 | 1.254998 | rno-miR-327 | 4.17E-05 | 0.782391 | rno-miR-465 | 0.006295 | 1.331138 |
| rno-miR-326 | 0.000824 | -1.24427 | rno-miR-148b-3p | 0.003987 | 0.493607 | rno-miR-451 | 0.00202 | -2.02923 | rno-miR-101b | 6.56E-05 | -0.80961 | rno-miR-3547 | 0.006479 | 1.630223 |
| rno-miR-207 | 0.000884 | 10.35882 | rno-miR-34c | 0.004686 | -0.74474 | rno-miR-872 | 0.002163 | -0.78616 | rno-miR-541 | 9.66E-05 | -13.1839 | rno-miR-652 | 0.006796 | -0.7383 |
| rno-miR-219-5p | 0.000998 | 11.03129 | rno-miR-101b | 0.004912 | -0.28229 | rno-miR-153 | 0.002259 | -2.24496 | rno-miR-369-5p | 0.000112 | -0.95307 | rno-miR-496 | 0.00721 | -0.93664 |
| rno-miR-668 | 0.001056 | -1.00808 | rno-miR-204 | 0.005628 | -0.75217 | rno-miR-3594-3p | 0.002623 | -1.57739 | rno-miR-494 | 0.000144 | 1.874271 | rno-miR-93 | 0.008224 | -0.9408 |
| rno-miR-672 | 0.00109 | 1.843793 | rno-miR-29c | 0.006084 | -0.81275 | rno-miR-665 | 0.002831 | -1.07068 | rno-let-7a-1 | 0.000189 | 2.039773 | rno-miR-339-3p | 0.008551 | 1.176405 |
| rno-miR-146a | 0.00119 | 1.448895 | rno-miR-340-5p | 0.006125 | 0.939928 | rno-miR-329 | 0.00314 | 0.959381 | rno-miR-30e | 0.000237 | -0.89537 | rno-miR-539 | 0.008914 | -1.16528 |
| rno-miR-215 | 0.00139 | 2.222185 | rno-miR-411 | 0.006464 | 0.991062 | rno-miR-628 | 0.003269 | 2.846071 | rno-miR-466d | 0.000434 | 2.56486 | rno-miR-192 | 0.009006 | -0.53439 |
| rno-miR-378 | 0.001555 | -0.6367 | rno-miR-3591 | 0.008219 | 0.503733 | rno-miR-187 | 0.003364 | 0.680947 | rno-miR-672 | 0.000709 | 0.802882 | rno-miR-19b | 0.009216 | -1.77119 |
| rno-miR-505 | 0.001991 | 0.573807 | rno-miR-3552 | 0.008321 | 0.954001 | rno-miR-455 | 0.003489 | -0.49967 | rno-miR-340-5p | 0.000757 | -0.77134 | rno-miR-376b-5p | 0.009852 | -1.03114 |
| rno-miR-3552 | 0.002202 | 0.890106 | rno-miR-667 | 0.008656 | 0.770531 | rno-miR-376a | 0.004934 | 1.065952 | rno-miR-295 | 0.000895 | 0.977283 | rno-miR-2985 | 0.010428 | 2.41551 |
| rno-miR-361 | 0.002206 | -0.93642 | rno-miR-708 | 0.00922 | -0.23765 | rno-miR-494 | 0.005099 | 1.781079 | rno-miR-369-3p | 0.000954 | -1.64814 | rno-miR-23b | 0.010529 | -0.29786 |
| rno-miR-7a-2 | 0.002365 | -1.17901 | rno-miR-365 | 0.009845 | 1.719326 | rno-miR-322 | 0.00551 | 2.095085 | rno-miR-484 | 0.000966 | 1.590507 | rno-miR-196b | 0.010577 | 1.771036 |
| rno-miR-345-5p | 0.002453 | -0.60231 | rno-miR-541 | 0.010031 | 0.597472 | rno-miR-369-5p | 0.006275 | -1.34282 | rno-miR-376b-5p | 0.001119 | -0.5717 | rno-miR-484 | 0.011594 | 1.271081 |
| rno-miR-434 | 0.002601 | -1.50775 | rno-miR-145 | 0.010068 | -0.70447 | rno-miR-3552 | 0.006949 | 1.045309 | rno-miR-874 | 0.001678 | -1.20234 | rno-miR-1 | 0.011796 | 1.225334 |
| rno-miR-290 | 0.002714 | 1.594176 | rno-miR-32 | 0.010107 | -0.9296 | rno-miR-295 | 0.007239 | 1.925773 | rno-miR-17-5p | 0.001739 | -0.1737 | rno-miR-325-5p | 0.011801 | -0.59414 |
| rno-miR-374 | 0.002729 | 1.213013 | rno-miR-208 | 0.010341 | -0.49579 | rno-miR-101a | 0.007314 | -0.71096 | rno-miR-291a-5p | 0.001772 | -1.40512 | rno-miR-410 | 0.011929 | -1.27826 |
| rno-miR-3593-3p | 0.003888 | 0.912062 | rno-miR-7b | 0.010525 | 1.676422 | rno-miR-325-5p | 0.00767 | 0.345292 | rno-miR-376c | 0.001784 | -0.9827 | rno-miR-664 | 0.012027 | -1.3631 |
| rno-miR-30a | 0.00398 | 1.406612 | rno-let-7f | 0.011028 | 0.570048 | rno-miR-3593-3p | 0.008662 | 0.733055 | rno-miR-297 | 0.001875 | 2.132592 | rno-miR-212 | 0.01236 | -0.53808 |
| rno-miR-376b-5p | 0.004214 | -0.61312 | rno-miR-3594-3p | 0.011106 | -0.71088 | rno-miR-3549 | 0.008804 | 1.353087 | rno-miR-3541 | 0.002168 | 2.423239 | rno-miR-382 | 0.012556 | 0.464989 |
| rno-miR-138-2 | 0.004351 | -0.4474 | rno-miR-494 | 0.011591 | 0.589636 | rno-miR-130a | 0.009363 | -0.60509 | rno-miR-675 | 0.002299 | -1.20322 | rno-miR-98 | 0.012696 | -0.22102 |
| rno-miR-194 | 0.004437 | 0.975612 | rno-miR-181b | 0.011644 | 0.527096 | rno-miR-9 | 0.010401 | -0.31986 | rno-miR-541 | 0.00246 | -0.26418 | rno-miR-674-3p | 0.012705 | -0.84699 |
| rno-miR-708 | 0.004524 | -0.50737 | rno-miR-9 | 0.011719 | 0.426885 | rno-miR-212 | 0.010441 | 0.551648 | rno-miR-2985 | 0.002472 | 2.019998 | rno-miR-22 | 0.012852 | -0.613 |
| rno-miR-541 | 0.004711 | 0.694375 | rno-miR-204 | 0.011935 | 0.483182 | rno-miR-93 | 0.010489 | 1.395421 | rno-miR-374 | 0.002478 | -0.66132 | rno-miR-195 | 0.012902 | -0.45101 |
| rno-miR-344b-5p | 0.004852 | -1.26909 | rno-miR-92a | 0.012356 | 0.525816 | rno-miR-3591 | 0.010758 | 1.357998 | rno-miR-322 | 0.002646 | -0.63753 | rno-miR-298 | 0.013279 | 1.547631 |
| rno-miR-134 | 0.005052 | -0.78026 | rno-miR-129-2 | 0.012372 | -0.74476 | rno-miR-181d | 0.010821 | -1.24707 | rno-miR-22 | 0.002957 | -0.66026 | rno-miR-122 | 0.013299 | 1.475554 |
| rno-miR-196b | 0.005237 | 1.046776 | rno-miR-455 | 0.012762 | -0.42292 | rno-miR-192 | 0.010878 | 0.513438 | rno-miR-3552 | 0.003064 | 0.541705 | rno-miR-126 | 0.014196 | -0.67038 |
| rno-miR-409-3p | 0.005435 | -0.28414 | rno-miR-338 | 0.012816 | 0.474084 | rno-miR-320 | 0.011206 | 1.884008 | rno-miR-34b | 0.003095 | 1.094634 | rno-miR-320 | 0.014299 | -0.55872 |
| rno-miR-329 | 0.00555 | -1.26763 | rno-miR-376a | 0.012954 | 0.529973 | rno-miR-221 | 0.0116 | 0.242714 | rno-miR-339-3p | 0.003377 | 0.973545 | rno-miR-764 | 0.014859 | 0.969683 |
| rno-miR-298 | 0.005884 | 1.287602 | rno-miR-139-3p | 0.01307 | 0.82799 | rno-miR-204 | 0.011685 | -1.05174 | rno-miR-325-5p | 0.003445 | -0.33356 | rno-miR-125a-3p | 0.015559 | 0.798486 |
| rno-miR-92a | 0.00721 | 0.684077 | rno-miR-325-5p | 0.013418 | -0.6394 | rno-miR-370 | 0.013161 | -1.33228 | rno-miR-296 | 0.00349 | 1.566458 | rno-miR-466d | 0.015697 | 3.330384 |
| rno-miR-181a-2 | 0.007511 | 1.475789 | rno-miR-3574 | 0.013497 | 1.113627 | rno-miR-21 | 0.013316 | -1.62573 | rno-miR-3588 | 0.003498 | -1.81969 | rno-miR-702-5p | 0.017074 | 1.128473 |
| rno-miR-543 | 0.007683 | -1.2652 | rno-miR-300-3p | 0.013524 | -0.44866 | rno-miR-598-3p | 0.01366 | -0.54814 | rno-miR-181a-2 | 0.003756 | 1.215901 | rno-miR-872 | 0.017348 | -0.77546 |
| rno-miR-7b | 0.008708 | 1.455591 | rno-miR-872 | 0.013633 | -0.40365 | rno-miR-652 | 0.013676 | -1.11531 | rno-miR-1249 | 0.003826 | 1.805086 | rno-miR-181c | 0.017477 | 0.449229 |
| rno-miR-135a | 0.009438 | -1.21904 | rno-miR-423 | 0.013758 | -0.78686 | rno-let-7b | 0.0137 | 0.971178 | rno-miR-326 | 0.003961 | 0.891332 | rno-miR-26b | 0.0176 | -0.40567 |
| rno-miR-411 | 0.010001 | -0.98954 | rno-miR-503 | 0.01471 | -0.9011 | rno-miR-434 | 0.014101 | -0.80798 | rno-miR-379 | 0.004121 | -0.78828 | rno-miR-652 | 0.017699 | 0.897737 |
| rno-miR-337 | 0.010045 | -0.88869 | rno-miR-210 | 0.015381 | -0.77523 | rno-miR-322 | 0.014609 | 1.498896 | rno-miR-325-3p | 0.004142 | -1.31805 | rno-miR-342-3p | 0.017772 | -0.4725 |
| rno-miR-151 | 0.010154 | -0.51022 | rno-miR-20a | 0.015388 | -0.53029 | rno-miR-154 | 0.014638 | -0.39435 | rno-miR-329 | 0.004343 | 0.403752 | rno-let-7b | 0.017949 | 0.727339 |
| rno-miR-338 | 0.010175 | 1.389807 | rno-miR-412 | 0.016394 | -1.77481 | rno-miR-223 | 0.015258 | 1.2148 | rno-miR-132 | 0.004993 | -1.14598 | rno-miR-3569 | 0.018091 | 1.221067 |
| rno-miR-34c | 0.010375 | 1.071076 | rno-miR-3573-3p | 0.017081 | 1.69769 | rno-miR-667 | 0.015838 | -1.4943 | rno-miR-664-1 | 0.005121 | -0.76359 | rno-miR-93 | 0.018709 | -0.35724 |
| rno-miR-15b | 0.010488 | 0.448593 | rno-miR-384-3p | 0.017209 | 0.693536 | rno-miR-668 | 0.016289 | -0.65411 | rno-let-7f-1 | 0.005282 | 2.189218 | rno-miR-16 | 0.018945 | -0.44196 |
| rno-miR-455 | 0.010647 | -0.38499 | rno-miR-568 | 0.01723 | 1.44412 | rno-miR-30e | 0.017805 | -1.47724 | rno-miR-496 | 0.005436 | -0.70402 | rno-miR-204 | 0.019222 | -0.41983 |
| rno-miR-223 | 0.010702 | 0.660332 | rno-miR-1 | 0.017587 | 1.287774 | rno-miR-383 | 0.018009 | -0.23863 | rno-miR-3557-3p | 0.005959 | -1.12862 | rno-miR-30e | 0.01939 | -0.23647 |
| rno-miR-300-3p | 0.010729 | -0.79176 | rno-miR-206 | 0.018308 | 1.714356 | rno-miR-296 | 0.018227 | 1.155502 | rno-miR-582 | 0.006065 | -1.15846 | rno-miR-295 | 0.019485 | 1.0957 |
| rno-miR-210 | 0.011165 | -0.75957 | rno-miR-337 | 0.018391 | -0.63803 | rno-miR-674-5p | 0.018468 | 0.505994 | rno-miR-872 | 0.006248 | -0.77301 | rno-miR-22 | 0.019722 | -0.13721 |
| rno-miR-30e | 0.011166 | 1.057734 | rno-miR-3593-3p | 0.018652 | 1.046588 | rno-miR-667 | 0.018474 | -0.99943 | rno-miR-3563-3p | 0.006392 | -1.17514 | rno-miR-352 | 0.019751 | -0.53148 |
| rno-miR-30b-5p | 0.011415 | 0.650187 | rno-miR-489 | 0.018769 | 1.199226 | rno-miR-215 | 0.01859 | 1.694058 | rno-miR-764 | 0.006539 | 0.831368 | rno-miR-378 | 0.020681 | -0.39057 |
| rno-miR-129-2 | 0.011648 | -0.7179 | rno-miR-182 | 0.018943 | 1.243974 | rno-miR-3594-5p | 0.018728 | -0.87093 | rno-miR-126 | 0.006581 | -1.98897 | rno-let-7f | 0.021112 | -0.56212 |
| rno-miR-652 | 0.011702 | 0.749358 | rno-miR-664 | 0.019275 | 1.447067 | rno-miR-191 | 0.018844 | -0.32388 | rno-miR-93 | 0.006656 | 2.242439 | rno-miR-23a | 0.021461 | 0.897594 |
| rno-miR-873 | 0.012299 | -1.70173 | rno-miR-382 | 0.019788 | 0.364538 | rno-miR-874 | 0.019184 | 0.832188 | rno-miR-361 | 0.006715 | 0.314415 | rno-miR-196c | 0.021555 | 1.420928 |
| rno-miR-106b | 0.012377 | -0.68353 | rno-miR-344a-3p | 0.019944 | -0.33801 | rno-miR-106b | 0.019494 | -0.87235 | rno-miR-290 | 0.007727 | -0.70433 | rno-miR-451 | 0.021772 | 1.866852 |
| rno-miR-181a | 0.01245 | -0.30061 | rno-let-7d | 0.020163 | 0.420761 | rno-miR-92a | 0.019575 | -0.74094 | rno-miR-28 | 0.008246 | -0.59369 | rno-miR-615 | 0.022368 | 0.745756 |
| rno-miR-188 | 0.012787 | 0.951533 | rno-miR-377 | 0.0205 | -1.56887 | rno-miR-103 | 0.019605 | 0.448949 | rno-miR-7a-1 | 0.008274 | -1.47343 | rno-miR-129-1 | 0.022493 | -1.21486 |
| rno-miR-369-3p | 0.012892 | 2.544034 | rno-miR-290 | 0.020623 | 1.314159 | rno-miR-128-2 | 0.019804 | -11.8611 | rno-miR-344b-2-3p | 0.008332 | -0.62808 | rno-miR-219-2-3p | 0.022824 | -0.14108 |
| rno-miR-320 | 0.013028 | 0.833259 | rno-miR-485 | 0.02144 | 0.253832 | rno-miR-99b | 0.019844 | -0.22389 | rno-miR-3555 | 0.008841 | 1.652384 | rno-miR-132 | 0.024065 | -0.52977 |
| rno-miR-325-5p | 0.013072 | -0.59004 | rno-miR-30c | 0.022092 | -0.30279 | rno-miR-342-3p | 0.021204 | -0.51785 | rno-miR-702-3p | 0.00894 | 0.793353 | rno-miR-137 | 0.024305 | -0.34963 |
| rno-miR-342-5p | 0.013156 | -0.98662 | rno-miR-466d | 0.023056 | -1.45169 | rno-miR-107 | 0.021372 | 0.386432 | rno-miR-153 | 0.009536 | -0.64695 | rno-miR-493 | 0.024565 | 1.084986 |
| rno-miR-539 | 0.013396 | 0.437616 | rno-miR-24 | 0.023127 | -0.3801 | rno-miR-222 | 0.021763 | 0.311459 | rno-miR-539 | 0.010323 | -0.65822 | rno-miR-434 | 0.024836 | -0.4698 |
| rno-miR-540 | 0.013734 | -1.13008 | rno-miR-187 | 0.023222 | 1.073099 | rno-miR-29c | 0.022281 | -1.16317 | rno-miR-328a | 0.01033 | -0.46062 | rno-miR-212 | 0.024845 | -0.40486 |
| rno-miR-29c | 0.014186 | -0.59938 | rno-miR-153 | 0.023527 | -0.82382 | rno-miR-138-1 | 0.023362 | 0.747412 | rno-miR-378 | 0.010855 | 0.197268 | rno-miR-711 | 0.024922 | 1.02176 |
| rno-miR-9 | 0.014463 | 0.260699 | rno-miR-146a | 0.023821 | 0.626407 | rno-miR-219-2-3p | 0.023957 | -0.38566 | rno-miR-133a | 0.011159 | -0.8833 | rno-miR-496 | 0.025585 | 1.003706 |
| rno-miR-329 | 0.014665 | -0.2877 | rno-miR-378 | 0.024128 | -0.35851 | rno-miR-496 | 0.02429 | -0.7683 | rno-miR-377 | 0.011261 | -0.5599 | rno-miR-3584-3p | 0.02606 | 1.301997 |
| rno-miR-383 | 0.014733 | -0.45254 | rno-miR-323 | 0.024145 | 0.503547 | rno-let-7a | 0.024666 | 0.260377 | rno-miR-433 | 0.01145 | 0.517363 | rno-miR-186 | 0.026208 | -0.72399 |
| rno-miR-222 | 0.015248 | 0.734193 | rno-miR-380 | 0.024211 | 0.718233 | rno-miR-3562 | 0.02487 | -0.17988 | rno-miR-34c | 0.011455 | 1.237287 | rno-miR-874 | 0.026256 | 0.360322 |
| rno-miR-485 | 0.015266 | -1.37357 | rno-miR-135a | 0.02449 | 0.994405 | rno-miR-465 | 0.025549 | 1.53137 | rno-miR-466c | 0.011867 | 0.60692 | rno-let-7e | 0.026478 | -0.55145 |
| rno-miR-664-2 | 0.015273 | -0.78786 | rno-miR-3597-5p | 0.024655 | 7.153379 | rno-miR-187 | 0.025596 | -0.57265 | rno-miR-99a | 0.011917 | -0.31081 | rno-miR-129-2 | 0.02659 | -0.81444 |
| rno-miR-3591 | 0.015338 | 0.786703 | rno-miR-21 | 0.024979 | -0.32385 | rno-miR-496 | 0.025861 | 1.098724 | rno-miR-493 | 0.012782 | 0.886179 | rno-miR-3591 | 0.027167 | 0.932865 |
| rno-let-7e | 0.015546 | 0.134515 | rno-miR-1224 | 0.025222 | -0.75886 | rno-miR-382 | 0.025942 | 1.736575 | rno-miR-485 | 0.013029 | 0.541279 | rno-miR-3594-5p | 0.028075 | 0.928802 |
| rno-let-7a-1 | 0.015664 | 0.944148 | rno-miR-327 | 0.02547 | -0.42082 | rno-let-7c | 0.026407 | 0.228872 | rno-miR-322 | 0.013081 | -0.64203 | rno-miR-3556a | 0.029677 | -1.18531 |
| rno-miR-27a | 0.016249 | 0.47581 | rno-miR-338 | 0.025732 | -0.65555 | rno-miR-146a | 0.027311 | 1.061442 | rno-miR-496 | 0.013612 | 1.144605 | rno-miR-181b | 0.030139 | -0.36275 |
| rno-miR-25 | 0.016257 | -0.30403 | rno-miR-127 | 0.025826 | -0.50883 | rno-miR-20a | 0.029118 | -0.52472 | rno-miR-15b | 0.013654 | -0.50204 | rno-miR-770 | 0.0308 | -0.65476 |
| rno-miR-34c | 0.016332 | -1.19064 | rno-miR-138-1 | 0.026514 | 0.422931 | rno-miR-323 | 0.03121 | 0.381338 | rno-miR-154 | 0.014014 | 0.157685 | rno-miR-181d | 0.031886 | -0.2696 |
| rno-miR-346 | 0.016443 | -0.35638 | rno-miR-543 | 0.026545 | -0.60482 | rno-miR-196b | 0.031866 | 0.758634 | rno-let-7f | 0.014106 | -0.35048 | rno-miR-1188-3p | 0.032447 | 0.829811 |
| rno-miR-382 | 0.017234 | 0.260191 | rno-miR-330 | 0.027178 | -0.35751 | rno-miR-138-2 | 0.033627 | 0.756658 | rno-miR-300-3p | 0.014111 | 0.411176 | rno-miR-490 | 0.033054 | -7.60189 |
| rno-miR-674-5p | 0.018517 | -0.26 | rno-miR-760-5p | 0.027314 | -1.18381 | rno-miR-764 | 0.033649 | 0.631755 | rno-miR-9 | 0.014576 | -0.24954 | rno-miR-27b | 0.033861 | -0.41125 |
| rno-miR-672 | 0.018865 | -1.54795 | rno-miR-326 | 0.027926 | -0.87486 | rno-miR-328b-3p | 0.033889 | -0.81922 | rno-miR-187 | 0.014781 | 0.356976 | rno-miR-148b-3p | 0.034017 | -1.22486 |
| rno-miR-434 | 0.018886 | -0.35558 | rno-miR-379 | 0.028086 | 1.539557 | rno-miR-376b-3p | 0.034142 | 0.47597 | rno-miR-221 | 0.015605 | -0.76661 | rno-miR-323 | 0.034704 | -1.24961 |
| rno-miR-3564 | 0.019115 | 0.814608 | rno-miR-223 | 0.028932 | 1.133729 | rno-miR-489 | 0.034375 | 1.117191 | rno-miR-125a-5p | 0.015918 | 0.146261 | rno-miR-322 | 0.036397 | -0.588 |
| rno-miR-101b | 0.01938 | -0.69412 | rno-miR-125b-5p | 0.029164 | -0.36183 | rno-miR-3568 | 0.034977 | 1.052991 | rno-miR-186 | 0.016168 | -0.42625 | rno-miR-678 | 0.036965 | 0.853909 |
| rno-miR-664 | 0.020035 | 0.305466 | rno-miR-540 | 0.029548 | -0.73559 | rno-miR-188 | 0.03581 | 0.39243 | rno-miR-664 | 0.016206 | -0.50708 | rno-miR-30a | 0.038164 | -2.46683 |
| rno-miR-381 | 0.020871 | -2.12637 | rno-miR-770 | 0.029711 | -0.38764 | rno-let-7i | 0.038138 | 0.267549 | rno-miR-3594-3p | 0.016313 | 1.190458 | rno-miR-124 | 0.040062 | -0.20594 |
| rno-miR-433 | 0.021954 | -0.57617 | rno-miR-3596b | 0.031594 | 1.328201 | rno-miR-369-3p | 0.038718 | -0.8817 | rno-miR-30c | 0.01638 | -0.19488 | rno-miR-25 | 0.040091 | -0.5421 |
| rno-miR-1188-5p | 0.022507 | -0.91671 | rno-miR-1193-3p | 0.032303 | -1.94406 | rno-miR-17-5p | 0.038986 | 0.347966 | rno-miR-672 | 0.016487 | -0.89805 | rno-miR-411 | 0.040382 | -0.73157 |
| rno-miR-678 | 0.023689 | 0.995602 | rno-miR-378 | 0.033671 | -1.08856 | rno-miR-224 | 0.039083 | 7.653355 | rno-miR-30a | 0.016555 | -0.17057 | rno-miR-27a | 0.040614 | -0.30727 |
| rno-miR-187 | 0.024177 | -0.18991 | rno-miR-873 | 0.034963 | -0.54542 | rno-miR-384-3p | 0.040711 | -1.30433 | rno-miR-219-2-3p | 0.017195 | -0.16795 | rno-miR-24 | 0.040716 | -0.17913 |
| rno-let-7b | 0.024677 | 0.950352 | rno-miR-30e | 0.036579 | 0.810817 | rno-miR-1 | 0.04275 | 2.523416 | rno-miR-208 | 0.017232 | -2.64551 | rno-miR-376c | 0.040847 | -0.97791 |
| rno-miR-874 | 0.025145 | 0.889259 | rno-miR-1249 | 0.037184 | 1.55309 | rno-miR-326 | 0.043237 | -0.46928 | rno-miR-146a | 0.01725 | -0.46555 | rno-miR-328a | 0.04216 | 0.369025 |
| rno-miR-29c | 0.026117 | 0.266775 | rno-miR-92a-2 | 0.039357 | -1.13931 | rno-miR-15b | 0.043735 | -0.62972 | rno-miR-212 | 0.017564 | -0.38603 | rno-miR-3582 | 0.043404 | 1.044215 |
| rno-miR-181d | 0.026738 | 1.374467 | rno-miR-410 | 0.039675 | 0.272781 | rno-miR-1188-5p | 0.046324 | -7.19552 | rno-miR-674-3p | 0.018316 | -0.31666 | rno-miR-300-3p | 0.044292 | -0.23301 |
| rno-miR-495 | 0.027153 | -0.36246 | rno-miR-374 | 0.039821 | 2.778641 | rno-miR-376c | 0.046648 | 2.679748 | rno-miR-187 | 0.018676 | 0.725362 | rno-miR-384-3p | 0.04469 | -0.43227 |
| rno-miR-667 | 0.027805 | -0.56617 | rno-let-7b | 0.040783 | 1.089008 | rno-miR-423 | 0.04668 | -0.3034 | rno-miR-338 | 0.01872 | -0.60397 | rno-miR-223 | 0.045236 | -0.85074 |
| rno-miR-150 | 0.028705 | -0.21831 | rno-miR-3547 | 0.041054 | 0.623815 | rno-miR-466b-1 | 0.047196 | -0.21189 | rno-miR-380 | 0.018832 | -0.73828 | rno-miR-15b | 0.046124 | -0.61027 |
| rno-miR-138-1 | 0.028939 | 0.451879 | rno-miR-628 | 0.041634 | 1.087186 | rno-miR-25 | 0.047433 | -0.22298 | rno-miR-465 | 0.018841 | 0.932209 | rno-miR-376b-3p | 0.046242 | -0.43406 |
| rno-miR-615 | 0.029241 | -0.97045 | rno-miR-3549 | 0.042544 | 1.130215 | rno-miR-23a | 0.047781 | 1.251825 | rno-miR-540 | 0.019162 | -0.65434 | rno-miR-34b | 0.046915 | 0.96093 |
| rno-miR-191 | 0.029521 | -0.24541 | rno-miR-322 | 0.042702 | 0.786652 | rno-miR-352 | 0.047895 | -0.36092 | rno-miR-151 | 0.019573 | 0.332143 | rno-miR-423 | 0.047186 | 1.047661 |
| rno-miR-667 | 0.030216 | 0.243097 | rno-miR-298 | 0.04362 | 0.642329 | rno-miR-379 | 0.048332 | 0.125636 | rno-miR-128 | 0.020045 | 0.164427 | rno-miR-26a | 0.048099 | -0.28512 |
| rno-miR-181b | 0.031846 | 0.369665 | rno-miR-3594-5p | 0.044037 | -0.46601 | rno-miR-1 | 0.049472 | 3.572176 | rno-miR-9 | 0.020317 | -0.23934 | rno-miR-328b-3p | 0.049643 | 0.652409 |
| rno-let-7i | 0.03199 | 0.069681 | rno-miR-543 | 0.044255 | -0.22023 | rno-miR-146b | 0.050328 | 0.275708 | rno-miR-361 | 0.020391 | 0.16676 | rno-miR-29c | 0.049662 | -0.24971 |
| rno-miR-3562 | 0.033361 | 0.403434 | rno-miR-151 | 0.046544 | -0.1458 | rno-miR-125b | 0.050545 | -1.02458 | rno-miR-1224 | 0.021093 | -0.23373 | rno-miR-128 | 0.050356 | -0.41318 |
| rno-miR-137 | 0.033634 | -0.74662 | rno-miR-200b | 0.047655 | -1.51404 | rno-miR-434 | 0.05153 | -0.21106 | rno-miR-375 | 0.021537 | 0.825819 | rno-miR-3568 | 0.050517 | 1.021253 |
| rno-miR-761 | 0.033838 | 1.104809 | rno-let-7b | 0.048677 | 0.40766 | rno-miR-31 | 0.055243 | 0.150122 | rno-miR-628 | 0.021592 | 1.454379 | rno-miR-125b-5p | 0.051531 | -0.2838 |
| rno-miR-194 | 0.033881 | 1.105353 | rno-miR-3568 | 0.049166 | -0.33511 | rno-miR-708 | 0.055868 | 0.6496 | rno-miR-129 | 0.021863 | 0.241524 | rno-miR-221 | 0.052864 | -2.34674 |
| rno-miR-1249 | 0.034009 | 1.376755 | rno-miR-22 | 0.049183 | 0.49225 | rno-miR-212 | 0.056045 | -0.53449 | rno-miR-495 | 0.022284 | -0.46064 | rno-miR-23a | 0.053511 | -0.38962 |
| rno-miR-702-3p | 0.034846 | 0.748926 | rno-miR-345-3p | 0.051033 | -0.92282 | rno-miR-411 | 0.056698 | -1.52109 | rno-miR-380 | 0.022813 | -0.80246 | rno-miR-485 | 0.053966 | -0.49101 |
| rno-miR-380 | 0.034949 | -0.49943 | rno-miR-191 | 0.051146 | 5.740729 | rno-miR-138 | 0.057227 | 0.28796 | rno-miR-702-5p | 0.022902 | 0.161504 | rno-miR-344b-5p | 0.055292 | -1.10001 |
| rno-miR-192 | 0.037126 | 0.546444 | rno-miR-341 | 0.051896 | -1.21794 | rno-miR-376b-5p | 0.059322 | 0.644976 | rno-miR-511 | 0.022919 | 0.96532 | rno-let-7d | 0.056456 | -0.3675 |
| rno-miR-466b-2 | 0.037144 | 0.340205 | rno-miR-409-5p | 0.052353 | -0.45735 | rno-miR-532-3p | 0.05961 | -6.5621 | rno-miR-7a | 0.023687 | -0.24182 | rno-miR-505 | 0.057584 | -0.8256 |
| rno-miR-382 | 0.03748 | 0.483115 | rno-miR-296 | 0.052654 | 0.942663 | rno-miR-702-3p | 0.060023 | 0.638841 | rno-miR-3550 | 0.024098 | 1.111206 | rno-miR-483 | 0.0576 | 1.708607 |
| rno-miR-152 | 0.037736 | 0.484868 | rno-miR-3564 | 0.053695 | 0.268787 | rno-miR-3596b | 0.060058 | -0.90153 | rno-miR-328b-3p | 0.024664 | 0.537062 | rno-miR-31 | 0.060291 | -0.37739 |
| rno-miR-1912-3p | 0.038045 | 3.895296 | rno-miR-300-5p | 0.055782 | -0.95062 | rno-miR-543 | 0.060185 | -1.44567 | rno-miR-3596c | 0.024795 | 2.16095 | rno-miR-532-5p | 0.060694 | -6.06507 |
| rno-miR-323 | 0.039519 | -0.37303 | rno-let-7a-1 | 0.057079 | 0.837249 | rno-miR-743a | 0.060611 | 6.301476 | rno-miR-206 | 0.024966 | 0.457683 | rno-miR-322 | 0.060792 | -0.82021 |
| rno-miR-218a | 0.040337 | 0.21026 | rno-miR-195 | 0.05769 | 0.463787 | rno-miR-504 | 0.060672 | -0.57676 | rno-miR-341 | 0.025158 | -0.73507 | rno-miR-325-3p | 0.061135 | -1.27396 |
| rno-let-7f | 0.041808 | 0.116331 | rno-miR-188 | 0.060025 | 0.53226 | rno-let-7d | 0.060935 | 0.682923 | rno-miR-127 | 0.025679 | 0.21316 | rno-miR-702-3p | 0.062514 | 1.018613 |
| rno-miR-322 | 0.042519 | 0.517305 | rno-miR-3541 | 0.0629 | 1.258338 | rno-miR-374 | 0.061822 | 0.916909 | rno-miR-761 | 0.025756 | 0.594291 | rno-miR-132 | 0.062683 | -0.99041 |
| rno-miR-30e | 0.043205 | 0.253311 | rno-miR-381 | 0.063547 | -2.75623 | rno-let-7a-2 | 0.063076 | -0.44207 | rno-miR-29b-2 | 0.025854 | 0.194214 | rno-miR-377 | 0.062856 | 0.544698 |
| rno-miR-29b | 0.043526 | 0.387149 | rno-miR-24-1 | 0.063766 | -1.02244 | rno-miR-664 | 0.063332 | -2.27104 | rno-miR-194 | 0.025869 | -0.94646 | rno-miR-3573-5p | 0.063581 | 0.816818 |
| rno-miR-29b-2 | 0.044428 | 0.836885 | rno-miR-296 | 0.065466 | -0.76065 | rno-miR-100 | 0.063445 | -0.26304 | rno-miR-181a | 0.026431 | 0.138944 | rno-miR-541 | 0.064876 | -0.47989 |
| rno-miR-3120 | 0.045623 | 1.260186 | rno-miR-674-3p | 0.070004 | -0.18012 | rno-miR-466d | 0.064739 | -1.90251 | rno-miR-665 | 0.027052 | 0.346754 | rno-miR-290 | 0.065733 | -0.41907 |
| rno-miR-101a | 0.046305 | -0.36079 | rno-miR-27a | 0.071988 | 0.216224 | rno-miR-101b | 0.066344 | -0.7998 | rno-miR-219-5p | 0.02725 | -1.10215 | rno-let-7a | 0.066516 | -0.13415 |
| rno-miR-431 | 0.04728 | -1.82796 | rno-miR-126 | 0.073886 | -0.20829 | rno-miR-122 | 0.066886 | 0.903676 | rno-miR-345-5p | 0.027273 | 0.342955 | rno-miR-541 | 0.067015 | -1.10401 |
| rno-miR-125a-5p | 0.048669 | -0.20039 | rno-miR-30a | 0.074297 | 0.557042 | rno-miR-1912-3p | 0.071028 | -1.23116 | rno-miR-204 | 0.027944 | -0.30874 | rno-miR-877 | 0.06891 | 0.603482 |
| rno-miR-126 | 0.05026 | 6.522241 | rno-miR-128 | 0.074663 | -0.28584 | rno-miR-487b | 0.071053 | -0.68914 | rno-miR-30a | 0.028061 | -0.4777 | rno-miR-350 | 0.068914 | -1.1119 |
| rno-miR-504 | 0.050653 | -0.74198 | rno-miR-99b | 0.075359 | 1.954108 | rno-miR-485 | 0.073402 | 0.429156 | rno-miR-3557-5p | 0.028162 | 1.384516 | rno-miR-3593-3p | 0.073039 | -0.62627 |
| rno-miR-1912-5p | 0.050887 | 2.197535 | rno-miR-137 | 0.076255 | -0.40176 | rno-miR-34a | 0.074132 | 0.16614 | rno-miR-207 | 0.029718 | 1.261016 | rno-miR-3562 | 0.076061 | 0.867663 |
| rno-miR-466b | 0.053257 | 0.676527 | rno-miR-365 | 0.076535 | -0.15658 | rno-miR-186 | 0.074742 | 0.957879 | rno-miR-3596a | 0.029722 | 0.53961 | rno-miR-193 | 0.076447 | -5.21783 |
| rno-miR-30c | 0.05456 | -0.17502 | rno-miR-539 | 0.076948 | 0.606532 | rno-miR-1249 | 0.074848 | 0.89852 | rno-miR-132 | 0.030048 | -0.20788 | rno-miR-667 | 0.077719 | 0.562128 |
| rno-miR-448 | 0.054567 | -0.42526 | rno-miR-764 | 0.077532 | -0.66867 | rno-miR-382 | 0.07499 | -0.17106 | rno-miR-3584-3p | 0.03043 | 0.517936 | rno-miR-99a | 0.078469 | -0.13806 |
| rno-miR-222 | 0.055097 | -0.15691 | rno-miR-299 | 0.079034 | -0.54924 | rno-miR-134 | 0.075356 | 0.216497 | rno-miR-3596b | 0.031416 | 0.541995 | rno-miR-382 | 0.080273 | -0.16995 |
| rno-miR-221 | 0.056654 | 0.363862 | rno-miR-505 | 0.079224 | 0.516336 | rno-miR-194 | 0.076338 | 0.66901 | rno-miR-298 | 0.031437 | 0.981903 | rno-miR-674-5p | 0.081036 | 0.317693 |
| rno-miR-3547 | 0.056755 | 0.697991 | rno-miR-3563-5p | 0.079272 | -0.49107 | rno-miR-433 | 0.078422 | 0.126166 | rno-miR-151 | 0.032345 | 0.169837 | rno-miR-30e | 0.083116 | -0.74046 |
| rno-miR-423 | 0.060265 | -0.2005 | rno-miR-3543 | 0.080238 | 4.419812 | rno-miR-26a | 0.078712 | 0.181002 | rno-miR-3593-3p | 0.032845 | -0.61372 | rno-miR-130a | 0.084747 | -0.28069 |
| rno-miR-130a | 0.063936 | -0.32561 | rno-miR-382 | 0.081486 | 0.559981 | rno-miR-3573-5p | 0.078799 | -1.10765 | rno-miR-3563-5p | 0.032868 | -0.32892 | rno-miR-361 | 0.08708 | 0.498661 |
| rno-miR-497 | 0.064406 | -0.58279 | rno-miR-154 | 0.081817 | 0.152261 | rno-miR-431 | 0.079536 | -0.72196 | rno-miR-107 | 0.032913 | 0.247814 | rno-let-7b | 0.087109 | -0.16617 |
| rno-miR-28 | 0.064739 | 1.011136 | rno-miR-133a | 0.082562 | -0.52657 | rno-miR-7b | 0.080374 | -0.29793 | rno-miR-27a | 0.03356 | -0.27327 | rno-miR-297 | 0.088519 | 5.306784 |
| rno-miR-664-1 | 0.064882 | -0.5745 | rno-miR-505 | 0.082896 | -0.41002 | rno-miR-92a-2 | 0.08354 | -1.67971 | rno-miR-27b | 0.03473 | -0.29904 | rno-miR-3594-3p | 0.090241 | 1.355946 |
| rno-miR-24-2 | 0.065023 | 0.793309 | rno-miR-532-5p | 0.086543 | -1.97221 | rno-miR-760-5p | 0.08568 | -0.41342 | rno-miR-324-3p | 0.034928 | 0.246859 | rno-miR-145 | 0.090426 | -1.03717 |
| rno-miR-22 | 0.066014 | -0.22802 | rno-miR-295 | 0.086919 | 0.519767 | rno-miR-380 | 0.085863 | 0.678859 | rno-miR-25 | 0.035159 | -0.31576 | rno-miR-345-5p | 0.090471 | 0.425322 |
| rno-miR-466c | 0.066095 | 0.253925 | rno-miR-381 | 0.088407 | 0.617381 | rno-miR-3573-3p | 0.086141 | 1.142579 | rno-miR-758 | 0.037086 | -0.49249 | rno-miR-7a-2 | 0.090851 | -0.49618 |
| rno-miR-326 | 0.071487 | -0.75536 | rno-let-7c | 0.091762 | 0.295057 | rno-miR-539 | 0.08663 | 1.134334 | rno-miR-423 | 0.037279 | 0.577037 | rno-miR-28 | 0.092321 | -0.50479 |
| rno-miR-543 | 0.072586 | -0.17869 | rno-miR-27b | 0.092402 | -1.18206 | rno-miR-3561-3p | 0.086642 | 0.870962 | rno-miR-133b | 0.037959 | -0.6892 | rno-miR-539 | 0.093332 | -0.17463 |
| rno-miR-3560 | 0.07275 | -1.47274 | rno-miR-324-3p | 0.093026 | -0.28206 | rno-miR-139-3p | 0.087608 | 0.473804 | rno-miR-199a-3p | 0.040956 | -1.31213 | rno-miR-207 | 0.09537 | 5.343944 |
| rno-miR-466b-1 | 0.073893 | 0.253224 | rno-miR-485 | 0.094856 | -0.20028 | rno-miR-466c | 0.095658 | -0.16006 | rno-miR-411 | 0.041396 | -0.40151 | rno-miR-3550 | 0.096379 | 4.607693 |
| rno-miR-204 | 0.075237 | 0.366578 | rno-miR-195 | 0.097518 | 0.198801 | rno-miR-135b | 0.096198 | -4.75026 | rno-miR-20a | 0.041884 | -0.45817 | rno-miR-320 | 0.09646 | 0.202658 |
| rno-miR-34b | 0.075628 | 0.748654 | rno-miR-496 | 0.097862 | -0.15162 | rno-miR-181c | 0.09694 | -0.29541 | rno-miR-141 | 0.044058 | 1.81752 | rno-miR-431 | 0.096974 | -1.80023 |
| rno-miR-760-3p | 0.07639 | 0.222544 | rno-miR-30a | 0.098788 | -0.18103 | rno-miR-410 | 0.097381 | -0.50428 | rno-miR-99b | 0.044753 | 0.176737 | rno-miR-873 | 0.097944 | -1.20691 |
| rno-miR-380 | 0.076938 | -0.47217 | rno-miR-134 | 0.10161 | 0.818475 | rno-miR-877 | 0.100367 | -0.34843 | rno-miR-221 | 0.04693 | -0.1595 | rno-miR-497 | 0.098023 | 0.281853 |
| rno-miR-199a-3p | 0.078181 | 0.843103 | rno-miR-185 | 0.104582 | -0.19807 | rno-miR-181b | 0.101911 | 0.151921 | rno-miR-93 | 0.047263 | 0.096476 | rno-let-7e | 0.099347 | -4.3705 |
| rno-miR-211 | 0.081066 | 1.801946 | rno-miR-3542 | 0.105229 | -0.51238 | rno-miR-361 | 0.108348 | -0.18722 | rno-miR-103 | 0.047456 | 0.198379 | rno-miR-369-5p | 0.101035 | -1.01558 |
| rno-miR-27b | 0.081588 | -0.15316 | rno-miR-361 | 0.105382 | -0.21508 | rno-miR-341 | 0.108993 | -4.23417 | rno-miR-488 | 0.047596 | -1.92259 | rno-miR-196a | 0.101088 | 0.766514 |
| rno-miR-24-1 | 0.083405 | -0.93683 | rno-miR-200b | 0.106242 | -0.57775 | rno-miR-3557-5p | 0.109808 | 5.694298 | rno-miR-125b-5p | 0.048075 | 0.156812 | rno-miR-375 | 0.101491 | 0.808359 |
| rno-miR-496 | 0.084097 | -0.16558 | rno-miR-124 | 0.113134 | -0.23444 | rno-miR-497 | 0.111396 | -0.24927 | rno-miR-7a-2 | 0.051469 | 0.366225 | rno-miR-127 | 0.102787 | -0.24285 |
| rno-miR-17-5p | 0.085743 | -0.32846 | rno-miR-344b-1-3p | 0.113276 | 0.320275 | rno-miR-664-2 | 0.111709 | -1.45495 | rno-miR-434 | 0.053133 | -0.11372 | rno-let-7i | 0.102942 | -0.23672 |
| rno-miR-124 | 0.087159 | -0.14285 | rno-miR-196c | 0.113379 | 0.481626 | rno-miR-2985 | 0.115493 | 2.082302 | rno-miR-30c-1 | 0.05395 | -0.60702 | rno-miR-758 | 0.104007 | -1.39554 |
| rno-miR-671 | 0.087337 | -0.69074 | rno-miR-342-3p | 0.11468 | 0.182445 | rno-miR-204 | 0.116337 | -0.2221 | rno-miR-674-5p | 0.05398 | 0.119223 | rno-miR-345-3p | 0.10449 | 0.818427 |
| rno-miR-135b | 0.08854 | 1.208175 | rno-miR-122 | 0.11622 | -4.2551 | rno-miR-409-3p | 0.122002 | 0.237586 | rno-miR-489 | 0.054451 | -0.61498 | rno-miR-99b | 0.108435 | -0.11941 |
| rno-miR-296 | 0.092173 | 0.868901 | rno-miR-210 | 0.119763 | 0.56845 | rno-miR-3557-3p | 0.12294 | 1.181537 | rno-miR-192 | 0.054783 | -0.6419 | rno-miR-3554 | 0.112408 | -0.76272 |
| rno-miR-212 | 0.092755 | 0.106621 | rno-miR-375 | 0.120358 | 1.525303 | rno-miR-182 | 0.123322 | 1.256648 | rno-miR-193 | 0.055271 | -0.49168 | rno-miR-3085 | 0.113933 | 0.440075 |
| rno-miR-675 | 0.093833 | 1.410586 | rno-miR-3582 | 0.12473 | 0.457839 | rno-miR-377 | 0.123514 | -5.46431 | rno-miR-347 | 0.056743 | 1.078608 | rno-miR-20b-5p | 0.114606 | -0.39649 |
| rno-miR-339-3p | 0.095639 | 0.312472 | rno-miR-672 | 0.127272 | -0.70892 | rno-miR-495 | 0.123644 | 0.176864 | rno-miR-487b | 0.057909 | 0.464937 | rno-miR-29c | 0.114701 | -0.35434 |
| rno-miR-488 | 0.096702 | 1.450118 | rno-miR-186 | 0.127914 | 0.415961 | rno-miR-106b | 0.123826 | -3.65094 | rno-miR-296 | 0.058303 | 0.452105 | rno-miR-490 | 0.115051 | -0.52715 |
| rno-miR-133a | 0.102357 | -0.41264 | rno-miR-615 | 0.132319 | 0.705635 | rno-miR-22 | 0.124647 | 0.247404 | rno-miR-124 | 0.059297 | 0.209325 | rno-miR-296 | 0.11744 | 0.981676 |
| rno-miR-652 | 0.102668 | -0.27172 | rno-miR-448 | 0.133605 | 1.108689 | rno-miR-350 | 0.129967 | -0.43689 | rno-miR-532-3p | 0.059453 | 0.604295 | rno-miR-329 | 0.119581 | -0.29532 |
| rno-miR-653 | 0.10306 | -4.44207 | rno-miR-434 | 0.135106 | 0.091718 | rno-miR-664-1 | 0.130697 | -3.4777 | rno-miR-411 | 0.05978 | -0.66342 | rno-miR-337 | 0.122427 | -0.43703 |
| rno-miR-292-5p | 0.104105 | 1.073019 | rno-miR-192 | 0.138256 | 0.510422 | rno-miR-330 | 0.131315 | 0.138682 | rno-miR-487b | 0.060147 | -0.14979 | rno-miR-455 | 0.122549 | -0.36831 |
| rno-miR-122 | 0.109182 | -4.43313 | rno-miR-140 | 0.141304 | 0.075519 | rno-miR-3541 | 0.132118 | 1.539 | rno-miR-125a-3p | 0.060801 | 0.390823 | rno-miR-17-5p | 0.124799 | -0.32948 |
| rno-miR-350 | 0.10962 | 0.332758 | rno-miR-664-2 | 0.143676 | 0.568143 | rno-miR-200b | 0.135477 | -0.8167 | rno-miR-139-5p | 0.062128 | -0.15784 | rno-miR-103-1 | 0.128594 | 1.323002 |
| rno-miR-383 | 0.111215 | -1.06286 | rno-miR-760-3p | 0.144007 | -0.39614 | rno-miR-3555 | 0.136383 | 1.406204 | rno-miR-505 | 0.062647 | -0.54717 | rno-miR-378 | 0.129429 | -0.9959 |
| rno-miR-378 | 0.113499 | -0.95706 | rno-miR-702-3p | 0.144472 | 0.658231 | rno-miR-3563-3p | 0.139922 | 0.638359 | rno-miR-135a | 0.062893 | -0.65921 | rno-miR-667 | 0.130085 | -0.50179 |
| rno-miR-193 | 0.116175 | 0.612709 | rno-miR-205 | 0.146465 | -4.52562 | rno-miR-3596a | 0.139974 | -4.39833 | rno-miR-3568 | 0.063331 | 0.431919 | rno-miR-298 | 0.132281 | 0.498416 |
| rno-miR-3588 | 0.117541 | 2.302118 | rno-miR-148b-5p | 0.149735 | -2.25189 | rno-miR-199a-3p | 0.143336 | -0.52742 | rno-miR-188 | 0.063875 | 0.571467 | rno-miR-3549 | 0.134091 | 3.20465 |
| rno-miR-503 | 0.121639 | -0.62542 | rno-miR-495 | 0.151336 | 0.79187 | rno-miR-541 | 0.144334 | 0.633554 | rno-miR-7b | 0.063935 | -0.66392 | rno-miR-151 | 0.135873 | -0.21356 |
| rno-miR-295 | 0.122011 | 0.866471 | rno-miR-3557-5p | 0.156887 | 0.837282 | rno-miR-124 | 0.145075 | -0.20549 | rno-let-7d | 0.064385 | -0.10887 | rno-miR-208 | 0.1364 | -0.17146 |
| rno-miR-758 | 0.127349 | -0.66784 | rno-miR-494 | 0.158049 | 0.508425 | rno-miR-376c | 0.145144 | 1.226224 | rno-miR-3547 | 0.0656 | 1.721868 | rno-miR-338 | 0.136613 | 0.459022 |
| rno-miR-3569 | 0.128487 | -0.78983 | rno-miR-138-2 | 0.158793 | -0.18972 | rno-miR-666 | 0.14801 | -2.78407 | rno-miR-100 | 0.06695 | -0.41598 | rno-miR-582 | 0.138955 | -1.75323 |
| rno-miR-122 | 0.131455 | 0.543688 | rno-miR-487b | 0.163084 | 0.106932 | rno-miR-7a-1 | 0.149052 | 1.439092 | rno-miR-872 | 0.067134 | -0.94361 | rno-miR-34a | 0.141053 | -0.33936 |
| rno-miR-129 | 0.133693 | 0.210679 | rno-miR-3563-3p | 0.16585 | 0.42667 | rno-miR-1949 | 0.151374 | 3.779183 | rno-miR-3556a | 0.068577 | -1.15279 | rno-miR-153 | 0.143508 | -2.6637 |
| rno-miR-145 | 0.136586 | 0.637815 | rno-miR-196b | 0.16784 | -0.60463 | rno-miR-493 | 0.151927 | 1.912642 | rno-miR-497 | 0.071502 | 0.398898 | rno-let-7c | 0.146362 | -0.20806 |
| rno-miR-489 | 0.138624 | 0.50281 | rno-miR-125b | 0.169138 | -0.32426 | rno-miR-1912-5p | 0.1611 | -0.70419 | rno-miR-92a | 0.077023 | -0.1772 | rno-miR-34c | 0.14779 | -0.56273 |
| rno-miR-3557-3p | 0.139423 | -0.53747 | rno-miR-337 | 0.174593 | -0.80306 | rno-miR-347 | 0.16272 | -2.26062 | rno-miR-483 | 0.077054 | 4.238514 | rno-miR-3557-5p | 0.149891 | -1.74854 |
| rno-miR-505 | 0.139544 | -0.87546 | rno-let-7a | 0.175759 | 0.233366 | rno-let-7e | 0.165255 | -1.45133 | rno-miR-298 | 0.078189 | 0.609397 | rno-miR-3555 | 0.151348 | -0.72876 |
| rno-miR-425 | 0.143793 | -2.05774 | rno-miR-292-5p | 0.176383 | 0.617375 | rno-miR-133a | 0.167026 | -0.56278 | rno-miR-543 | 0.078828 | -0.39269 | rno-miR-153 | 0.151614 | -0.82133 |
| rno-miR-107 | 0.144891 | -0.13675 | rno-miR-212 | 0.176718 | -0.1862 | rno-miR-490 | 0.168819 | 1.618246 | rno-miR-152 | 0.085899 | 1.703625 | rno-miR-532-3p | 0.152974 | 0.636197 |
| rno-miR-1 | 0.146929 | 0.763944 | rno-miR-376c | 0.178399 | 1.767936 | rno-miR-340-5p | 0.170282 | 3.562579 | rno-miR-451 | 0.095477 | 0.204588 | rno-miR-127 | 0.153836 | -0.54976 |
| rno-miR-19b | 0.147588 | 0.307059 | rno-miR-678 | 0.179632 | 0.482226 | rno-miR-485 | 0.170926 | -0.19122 | rno-miR-671 | 0.096653 | 0.442132 | rno-miR-21 | 0.154962 | 2.460662 |
| rno-miR-1 | 0.149347 | 0.776343 | rno-miR-107 | 0.181231 | -1.40129 | rno-miR-327 | 0.1763 | 0.17476 | rno-miR-547 | 0.098653 | 0.536864 | rno-miR-193 | 0.155068 | -1.0153 |
| rno-miR-125b-5p | 0.150373 | -0.10261 | rno-miR-412 | 0.181886 | -0.3025 | rno-miR-761 | 0.176855 | 3.388673 | rno-miR-292-5p | 0.102638 | 0.459796 | rno-miR-425 | 0.156629 | -0.18108 |
| rno-let-7d | 0.150458 | -0.0315 | rno-miR-150 | 0.1833 | -0.07093 | rno-miR-1188-3p | 0.181508 | -0.47963 | rno-let-7b | 0.102935 | 0.365569 | rno-miR-433 | 0.164036 | -0.1309 |
| rno-miR-702-5p | 0.157379 | 0.628396 | rno-miR-299 | 0.184068 | -1.13928 | rno-miR-194 | 0.183518 | 0.520932 | rno-miR-181a-1 | 0.103139 | -2.15224 | rno-miR-324-3p | 0.164608 | -0.23699 |
| rno-miR-3541 | 0.162146 | 0.405189 | rno-miR-191 | 0.184688 | -0.14574 | rno-miR-3584-5p | 0.184825 | 0.176119 | rno-miR-211 | 0.103536 | 0.591379 | rno-miR-347 | 0.165579 | 1.431578 |
| rno-miR-3568 | 0.162371 | -0.24937 | rno-miR-221 | 0.185033 | -0.55998 | rno-miR-132 | 0.191614 | -0.17578 | rno-miR-877 | 0.104507 | 0.266095 | rno-miR-708 | 0.165582 | -2.08366 |
| rno-let-7c | 0.167758 | -0.08986 | rno-miR-19b | 0.185042 | -0.17794 | rno-miR-297 | 0.192599 | -1.66312 | rno-miR-16 | 0.105295 | 1.036933 | rno-miR-130b | 0.166545 | -1.11613 |
| rno-miR-465 | 0.168827 | -0.57633 | rno-miR-425 | 0.185074 | -1.5469 | rno-miR-93 | 0.196664 | -0.10593 | rno-miR-433 | 0.108199 | -0.38056 | rno-miR-872 | 0.167937 | -1.11128 |
| rno-miR-409-5p | 0.179467 | -1.19309 | rno-miR-542-5p | 0.185174 | -3.31432 | rno-miR-29b-2 | 0.198507 | -1.01471 | rno-miR-3573-3p | 0.117095 | 2.67943 | rno-miR-370 | 0.172623 | 1.645953 |
| rno-miR-30c-1 | 0.182059 | 0.82463 | rno-miR-122 | 0.186063 | -0.38192 | rno-miR-127 | 0.201048 | -0.09286 | rno-miR-365 | 0.118133 | 0.643132 | rno-miR-133b | 0.172629 | 0.473848 |
| rno-miR-935 | 0.182832 | -1.48197 | rno-miR-9 | 0.193206 | -0.12834 | rno-miR-298 | 0.202491 | 1.324729 | rno-miR-153 | 0.119811 | -0.46309 | rno-miR-500 | 0.17316 | -0.74195 |
| rno-miR-30c-2 | 0.187741 | 0.289191 | rno-miR-551b | 0.193622 | 2.707563 | rno-miR-3120 | 0.203122 | 0.791937 | rno-miR-664-2 | 0.120707 | -0.64501 | rno-miR-379 | 0.181028 | -1.84566 |
| rno-miR-139-3p | 0.189503 | 0.374421 | rno-miR-671 | 0.199202 | 0.902517 | rno-miR-151 | 0.204452 | 0.138321 | rno-miR-1 | 0.124125 | 1.110081 | rno-miR-140 | 0.184418 | -0.10065 |
| rno-let-7f-2 | 0.189912 | 1.855434 | rno-miR-181c | 0.202149 | -0.44281 | rno-miR-134 | 0.206796 | -0.89204 | rno-miR-382 | 0.12422 | -0.07207 | rno-miR-292-5p | 0.185293 | 0.412198 |
| rno-miR-665 | 0.199727 | -0.11118 | rno-miR-28 | 0.208067 | -1.76618 | rno-miR-127 | 0.20856 | -1.05179 | rno-miR-448 | 0.128044 | -0.13337 | rno-miR-412 | 0.185893 | -1.08657 |
| rno-miR-370 | 0.199993 | 0.683197 | rno-miR-490 | 0.212006 | 0.477216 | rno-miR-99b | 0.208988 | -1.42066 | rno-miR-191 | 0.129326 | 0.715569 | rno-miR-106b | 0.196715 | -1.53461 |
| rno-miR-30a | 0.200883 | 0.105549 | rno-miR-199a-3p | 0.212365 | -1.13401 | rno-miR-448 | 0.214447 | -0.46086 | rno-miR-505 | 0.129668 | -0.45167 | rno-miR-760-3p | 0.197038 | 0.309501 |
| rno-miR-3573-3p | 0.208993 | 0.336341 | rno-miR-376c | 0.213783 | 2.727547 | rno-miR-370 | 0.214547 | -3.33899 | rno-miR-1188-5p | 0.13006 | -2.62688 | rno-miR-30a | 0.198788 | -0.10536 |
| rno-miR-3594-3p | 0.209528 | -0.7937 | rno-miR-370 | 0.217164 | 1.019075 | rno-miR-760-3p | 0.214862 | 0.25861 | rno-miR-210 | 0.133334 | 0.377745 | rno-miR-138 | 0.200687 | -0.12649 |
| rno-miR-487b | 0.211003 | 0.786693 | rno-miR-501 | 0.219628 | -1.91473 | rno-miR-338 | 0.22101 | -0.59801 | rno-miR-381 | 0.134481 | 0.151344 | rno-miR-374 | 0.201724 | -0.5208 |
| rno-miR-132 | 0.219823 | -0.10205 | rno-miR-598-5p | 0.223122 | 1.174024 | rno-miR-3586-5p | 0.222927 | -3.10823 | rno-miR-652 | 0.134649 | -0.1148 | rno-miR-7a-1 | 0.201778 | -0.19641 |
| rno-miR-221 | 0.22215 | -0.08266 | rno-miR-3596a | 0.226813 | -0.23814 | rno-let-7f | 0.222932 | 0.312475 | rno-miR-667 | 0.136858 | -0.34095 | rno-miR-331 | 0.202352 | 1.721059 |
| rno-miR-3572 | 0.222949 | -2.41049 | rno-miR-181a-1 | 0.23008 | -2.21105 | rno-miR-379 | 0.224927 | 0.5227 | rno-miR-148b-3p | 0.139674 | -0.13312 | rno-miR-628 | 0.203137 | 0.378285 |
| rno-miR-185 | 0.231849 | -0.06356 | rno-miR-433 | 0.233954 | 0.102317 | rno-miR-361 | 0.226346 | -0.07145 | rno-miR-27b | 0.141589 | -0.88328 | rno-miR-409-5p | 0.204986 | -1.1732 |
| rno-miR-299 | 0.233291 | 0.278077 | rno-miR-3584-3p | 0.234068 | 0.677354 | rno-miR-148b-3p | 0.227932 | 1.152765 | rno-miR-337 | 0.14354 | 0.07403 | rno-miR-150 | 0.20503 | -1.23721 |
| rno-miR-874 | 0.236247 | -0.88732 | rno-miR-666 | 0.237462 | 2.136553 | rno-miR-615 | 0.229159 | -3.10468 | rno-miR-1188-3p | 0.14635 | 0.53999 | rno-miR-343 | 0.209243 | 2.652923 |
| rno-miR-31 | 0.244667 | 1.019592 | rno-miR-132 | 0.238582 | 0.17316 | rno-miR-344b-5p | 0.243345 | 1.145807 | rno-miR-376c | 0.147502 | -0.31072 | rno-miR-30c-1 | 0.211034 | 1.414884 |
| rno-miR-193 | 0.245715 | 0.612176 | rno-miR-218a | 0.240854 | -0.15301 | rno-miR-125a-3p | 0.247029 | -0.44277 | rno-miR-370 | 0.149341 | -0.42852 | rno-miR-3586-5p | 0.217315 | 3.180563 |
| rno-miR-666 | 0.246277 | -2.49913 | rno-miR-3546 | 0.242723 | 2.437899 | rno-miR-466b-2 | 0.2503 | 0.056634 | rno-miR-331 | 0.14963 | 0.128572 | rno-miR-760-5p | 0.218453 | 0.643927 |
| rno-miR-455 | 0.247311 | -11.4245 | rno-miR-339-3p | 0.243231 | 0.422145 | rno-miR-500 | 0.25312 | -0.3559 | rno-miR-708 | 0.15162 | -3.47635 | rno-miR-154 | 0.221736 | -0.16637 |
| rno-miR-450a | 0.251584 | -11.2495 | rno-miR-872 | 0.25047 | 0.733353 | rno-miR-145 | 0.257306 | 0.091991 | rno-miR-24 | 0.15286 | 0.195046 | rno-miR-326 | 0.227505 | 0.228068 |
| rno-miR-127 | 0.254403 | -0.07087 | rno-miR-294 | 0.250668 | 0.41071 | rno-miR-24-2 | 0.264282 | 0.667938 | rno-miR-409-5p | 0.163188 | -0.47787 | rno-miR-194 | 0.228166 | -1.19449 |
| rno-miR-125b | 0.255864 | 0.189824 | rno-miR-3085 | 0.25236 | 1.640002 | rno-miR-339-3p | 0.265392 | 0.434295 | rno-miR-140 | 0.164349 | 0.15532 | rno-miR-194 | 0.230124 | -0.5407 |
| rno-miR-185 | 0.260576 | 10.21051 | rno-miR-375 | 0.25369 | 0.8094 | rno-miR-3577 | 0.266465 | -2.52091 | rno-miR-204 | 0.166744 | 0.366985 | rno-miR-501 | 0.232101 | -0.54642 |
| rno-miR-153 | 0.262504 | 0.498777 | rno-miR-370 | 0.25699 | 0.58881 | rno-miR-23b | 0.267396 | 0.828866 | rno-miR-194 | 0.169222 | -0.81066 | rno-miR-294 | 0.232397 | 0.919856 |
| rno-miR-376a | 0.265653 | -1.4675 | rno-miR-320 | 0.257644 | 0.335885 | rno-miR-488 | 0.268966 | -0.46949 | rno-miR-3569 | 0.16995 | 0.404469 | rno-miR-3546 | 0.234885 | 0.796086 |
| rno-miR-361 | 0.266928 | -0.09367 | rno-miR-181a-2 | 0.26155 | -0.5999 | rno-miR-874 | 0.279094 | -2.09427 | rno-miR-24-2 | 0.170974 | -0.26188 | rno-miR-376a | 0.237906 | -0.30399 |
| rno-miR-20b-5p | 0.269609 | 2.186193 | rno-miR-99b | 0.262081 | -0.06718 | rno-miR-300-3p | 0.287366 | -0.1481 | rno-let-7d | 0.171814 | 0.114627 | rno-miR-434 | 0.238384 | -0.44529 |
| rno-miR-9 | 0.275504 | 0.086594 | rno-miR-2985 | 0.267471 | 0.567993 | rno-miR-378 | 0.290024 | -0.43385 | rno-miR-20b-5p | 0.172909 | -0.64089 | rno-miR-330 | 0.239059 | 0.551124 |
| rno-miR-369-5p | 0.289275 | -0.09769 | rno-miR-15b | 0.268622 | -0.13474 | rno-miR-3550 | 0.290149 | -2.12561 | rno-miR-344b-5p | 0.17299 | -0.11684 | rno-miR-181c | 0.254584 | -1.50402 |
| rno-miR-27b | 0.290582 | -1.46333 | rno-miR-448 | 0.269217 | -0.23069 | rno-miR-383 | 0.294056 | -2.19535 | rno-miR-708 | 0.182787 | 0.115061 | rno-miR-340-5p | 0.257298 | -1.0075 |
| rno-miR-377 | 0.293727 | -1.97971 | rno-miR-326 | 0.270435 | -0.73751 | rno-miR-412 | 0.295364 | -2.65491 | rno-miR-223 | 0.186767 | 0.261179 | rno-miR-106b | 0.261181 | -0.24387 |
| rno-miR-3551-5p | 0.296542 | 0.942533 | rno-miR-3586-5p | 0.272078 | 0.642292 | rno-miR-30c-1 | 0.296487 | -1.50607 | rno-miR-675 | 0.190804 | -3.66062 | rno-miR-138-1 | 0.261825 | 0.107835 |
| rno-let-7b | 0.30333 | -0.04566 | rno-miR-652 | 0.274246 | 0.092265 | rno-miR-136 | 0.299333 | 1.879582 | rno-miR-22 | 0.196745 | -0.07997 | rno-miR-873 | 0.264761 | -2.28483 |
| rno-miR-23a | 0.312794 | -0.0449 | rno-miR-301a | 0.275808 | -1.30455 | rno-miR-345-5p | 0.302781 | 0.231329 | rno-miR-138-2 | 0.197589 | 0.17825 | rno-miR-185 | 0.275522 | -0.04994 |
| rno-miR-125a-3p | 0.315262 | 0.258024 | rno-miR-3557-3p | 0.285759 | 1.869924 | rno-miR-3547 | 0.302878 | -0.55666 | rno-miR-18a | 0.197881 | 0.751188 | rno-miR-540 | 0.281611 | -1.06228 |
| rno-miR-381 | 0.320987 | -0.27712 | rno-miR-1949 | 0.286808 | -0.53802 | rno-miR-381 | 0.306794 | 1.997134 | rno-miR-193 | 0.202295 | -1.59544 | rno-miR-3588 | 0.282381 | -2.08299 |
| rno-miR-151 | 0.323009 | -0.08199 | rno-miR-200c | 0.288641 | -2.17308 | rno-miR-185 | 0.308412 | -0.02934 | rno-miR-125b | 0.208561 | -0.09738 | rno-miR-3563-5p | 0.283554 | -1.02906 |
| rno-miR-136 | 0.324389 | 0.946149 | rno-miR-297 | 0.288699 | -2.33914 | rno-miR-181d | 0.311554 | -0.84938 | rno-miR-490 | 0.21113 | -0.73512 | rno-miR-126 | 0.285754 | -0.55629 |
| rno-let-7a | 0.330645 | 0.068965 | rno-miR-487b | 0.290665 | 1.95129 | rno-miR-872 | 0.311569 | 1.891391 | rno-miR-760-3p | 0.21751 | 0.082447 | rno-miR-344a-3p | 0.286816 | -0.18472 |
| rno-miR-136 | 0.334177 | 0.881398 | rno-miR-347 | 0.292882 | -0.63561 | rno-miR-708 | 0.315363 | 1.836435 | rno-miR-19b | 0.229387 | -1.51811 | rno-miR-761 | 0.287812 | 0.754031 |
| rno-miR-3085 | 0.334691 | 0.56356 | rno-miR-653 | 0.29563 | 1.791711 | rno-miR-3553 | 0.320167 | 1.567139 | rno-miR-873 | 0.231901 | -0.37046 | rno-miR-107 | 0.290325 | -0.13327 |
| rno-miR-582 | 0.335552 | -1.61594 | rno-miR-320 | 0.295719 | -0.31879 | rno-miR-222 | 0.327762 | 1.166873 | rno-miR-3542 | 0.232007 | 2.752423 | rno-miR-129 | 0.295619 | 0.086533 |
| rno-miR-23b | 0.336284 | -0.74426 | rno-miR-329 | 0.300729 | 0.073869 | rno-miR-30a | 0.329411 | -1.07448 | rno-miR-134 | 0.233684 | 0.060782 | rno-miR-568 | 0.296527 | 0.068926 |
| rno-miR-551b | 0.337811 | -0.75263 | rno-miR-214 | 0.305487 | -0.08245 | rno-miR-195 | 0.33189 | 0.090935 | rno-miR-678 | 0.241138 | -0.24497 | rno-miR-191 | 0.296717 | 0.602137 |
| rno-miR-493 | 0.338857 | -1.11939 | rno-miR-547 | 0.305529 | -0.412 | rno-miR-152 | 0.332492 | 0.720133 | rno-miR-143 | 0.243522 | 0.088834 | rno-miR-501 | 0.296913 | -1.65116 |
| rno-miR-598-3p | 0.341465 | 0.099955 | rno-miR-30c-2 | 0.306791 | -0.23434 | rno-miR-325-3p | 0.335113 | -0.2206 | rno-miR-378 | 0.245273 | 0.436712 | rno-miR-181a | 0.304791 | -0.09559 |
| rno-miR-3563-5p | 0.350909 | -0.2547 | rno-miR-1188-5p | 0.307138 | -1.98036 | rno-miR-425 | 0.336612 | -1.5633 | rno-miR-668 | 0.250262 | -0.20523 | rno-miR-152 | 0.313027 | -1.58682 |
| rno-miR-322 | 0.355977 | 0.449387 | rno-miR-881 | 0.307827 | -1.28142 | rno-miR-34c | 0.336774 | -2.61694 | rno-miR-330 | 0.255228 | 0.262513 | rno-miR-222 | 0.323208 | -1.60742 |
| rno-miR-23b | 0.371433 | -0.05441 | rno-miR-483 | 0.31183 | 1.620976 | rno-miR-411 | 0.339652 | 0.444873 | rno-miR-29a | 0.264568 | 0.058067 | rno-miR-503 | 0.323523 | -0.79388 |
| rno-miR-3556a | 0.385223 | 0.291173 | rno-miR-181c | 0.312757 | 0.144109 | rno-miR-30c-2 | 0.339996 | -1.48068 | rno-miR-343 | 0.264651 | 2.306327 | rno-miR-380 | 0.324139 | -1.56284 |
| rno-miR-3558-5p | 0.385266 | -1.13498 | rno-miR-194 | 0.31318 | 0.254599 | rno-miR-547 | 0.343496 | -1.45278 | rno-miR-375 | 0.274402 | -0.41928 | rno-miR-381 | 0.325724 | -0.31924 |
| rno-miR-628 | 0.385716 | 0.679356 | rno-miR-26a | 0.31547 | -1.76821 | rno-miR-191 | 0.343733 | 0.715381 | rno-miR-185 | 0.279525 | 1.911679 | rno-miR-3596c | 0.326321 | 1.397348 |
| rno-miR-186 | 0.393615 | -0.1825 | rno-miR-132 | 0.321364 | -0.13071 | rno-miR-505 | 0.359931 | -0.21755 | rno-miR-136 | 0.284894 | -0.48918 | rno-let-7a-1 | 0.326564 | -0.15075 |
| rno-miR-3594-5p | 0.395942 | -0.18244 | rno-miR-667 | 0.322584 | 0.211241 | rno-miR-150 | 0.363099 | 0.198559 | rno-miR-412 | 0.300311 | -0.37583 | rno-miR-17-1-3p | 0.326631 | -1.54568 |
| rno-miR-26a | 0.399246 | 0.052766 | rno-miR-295 | 0.335161 | -1.70937 | rno-miR-34b | 0.363919 | -1.33582 | rno-miR-200b | 0.310359 | -0.8289 | rno-miR-7a | 0.327235 | 0.108253 |
| rno-miR-711 | 0.405052 | -0.35089 | rno-miR-3569 | 0.336268 | -0.4641 | rno-miR-412 | 0.372445 | -1.19172 | rno-miR-350 | 0.314905 | 0.239181 | rno-miR-182 | 0.327938 | -0.15092 |
| rno-miR-675 | 0.415902 | 0.635724 | rno-miR-532-3p | 0.341574 | -1.37729 | rno-miR-344a-3p | 0.377339 | -0.04466 | rno-miR-345-3p | 0.318879 | 0.271611 | rno-miR-323 | 0.334065 | -0.31067 |
| rno-miR-3558-3p | 0.419302 | -0.72576 | rno-let-7f-1 | 0.346268 | -0.27714 | rno-miR-423 | 0.380442 | -0.78596 | rno-miR-181c | 0.324455 | 0.084005 | rno-miR-409-3p | 0.336327 | 0.189199 |
| rno-miR-872 | 0.419922 | 0.138455 | rno-miR-666 | 0.347481 | 1.260206 | rno-miR-375 | 0.384247 | -1.05047 | rno-let-7c | 0.328944 | 0.084053 | rno-miR-335 | 0.338925 | 0.310145 |
| rno-miR-224 | 0.421817 | -0.52054 | rno-miR-103-1 | 0.348022 | -0.10799 | rno-miR-409-5p | 0.384818 | -1.04836 | rno-miR-3561-3p | 0.333495 | 0.669588 | rno-miR-341 | 0.344825 | 0.221836 |
| rno-miR-2985 | 0.422074 | 0.750721 | rno-miR-493 | 0.357827 | 0.496409 | rno-miR-92b | 0.386838 | -0.08099 | rno-miR-344b-1-3p | 0.336219 | -0.16457 | rno-miR-206 | 0.345204 | -0.07232 |
| rno-miR-412 | 0.424045 | -0.45273 | rno-miR-194 | 0.358292 | 0.300457 | rno-miR-380 | 0.401696 | -0.86879 | rno-miR-34c | 0.340429 | 0.102142 | rno-miR-210 | 0.345265 | 0.328837 |
| rno-miR-3555 | 0.426973 | -0.52954 | rno-miR-496 | 0.364299 | -0.26818 | rno-miR-211 | 0.403696 | 0.187656 | rno-miR-215 | 0.345287 | 0.163584 | rno-miR-369-3p | 0.345937 | -0.34591 |
| rno-miR-1188-3p | 0.433641 | 0.223129 | rno-miR-409-3p | 0.366908 | -0.10013 | rno-miR-181a-2 | 0.410275 | 0.567669 | rno-miR-652 | 0.350311 | -0.07324 | rno-miR-134 | 0.348576 | -0.05882 |
| rno-miR-3596b | 0.438112 | 0.480053 | rno-miR-127 | 0.367429 | -0.09623 | rno-miR-296 | 0.413333 | -0.6864 | rno-miR-3584-5p | 0.351372 | 0.109484 | rno-miR-375 | 0.348846 | -0.5296 |
| rno-miR-3553 | 0.439057 | -0.76478 | rno-miR-465 | 0.368807 | -0.14217 | rno-miR-336 | 0.416169 | 11.40615 | rno-miR-24-1 | 0.355978 | -0.42717 | rno-miR-379 | 0.355427 | 0.091251 |
| rno-miR-466d | 0.439142 | 0.381498 | rno-miR-133b | 0.369764 | -0.21609 | rno-miR-193 | 0.420415 | 0.322832 | rno-miR-329 | 0.361288 | 0.065873 | rno-let-7d | 0.356939 | 0.162373 |
| rno-miR-345-3p | 0.443143 | 0.375988 | rno-miR-200a | 0.382746 | -1.44942 | rno-miR-3572 | 0.421183 | 0.660981 | rno-miR-152 | 0.361517 | 0.11709 | rno-miR-329 | 0.360255 | -0.19387 |
| rno-miR-490 | 0.454077 | 0.180015 | rno-miR-3120 | 0.395121 | 0.944937 | rno-miR-3569 | 0.424572 | -0.90928 | rno-miR-26a | 0.381621 | 0.098751 | rno-miR-24-1 | 0.363953 | -1.02045 |
| rno-let-7a-2 | 0.456397 | 0.708394 | rno-miR-3573-5p | 0.398375 | 0.276739 | rno-miR-878 | 0.429017 | -12.7691 | rno-miR-137 | 0.383071 | 0.169281 | rno-miR-24-2 | 0.367386 | -0.34995 |
| rno-miR-106b | 0.467206 | 0.124348 | rno-miR-652 | 0.400572 | 0.042665 | rno-miR-326 | 0.438356 | 0.30482 | rno-miR-145 | 0.384497 | -0.42273 | rno-miR-708 | 0.385484 | -0.15773 |
| rno-miR-3557-5p | 0.470718 | -0.24117 | rno-miR-92b | 0.403478 | 0.04034 | rno-miR-103-1 | 0.440246 | 0.224517 | rno-miR-434 | 0.387382 | -0.09486 | rno-miR-383 | 0.397694 | -0.63047 |
| rno-miR-3582 | 0.471447 | 0.253467 | rno-miR-874 | 0.405735 | -0.86601 | rno-miR-3556a | 0.444335 | 0.364519 | rno-miR-26b | 0.393482 | -2.43001 | rno-miR-3596b | 0.400693 | 0.358938 |
| rno-miR-294 | 0.471882 | 0.283366 | rno-miR-222 | 0.409544 | -0.05406 | rno-miR-770 | 0.445497 | -0.04582 | rno-miR-23a | 0.397574 | -0.10492 | rno-miR-764 | 0.401297 | -0.7189 |
| rno-miR-99b | 0.473458 | -0.3614 | rno-miR-761 | 0.415148 | 0.60875 | rno-miR-483 | 0.459184 | 0.074586 | rno-miR-30c-2 | 0.399274 | -0.16629 | rno-miR-330 | 0.408052 | 0.115593 |
| rno-miR-484 | 0.483803 | 0.221466 | rno-miR-433 | 0.416627 | 0.250852 | rno-miR-291a-5p | 0.472126 | -0.22385 | rno-miR-181c | 0.447979 | 0.436361 | rno-miR-3558-5p | 0.412335 | 0.62154 |
| rno-miR-349 | 0.494878 | -2.06374 | rno-miR-582 | 0.424856 | 0.313581 | rno-miR-210 | 0.473771 | 0.269619 | rno-miR-323 | 0.449017 | -1.77101 | rno-miR-489 | 0.427971 | 0.183584 |
| rno-miR-22 | 0.498341 | 0.147717 | rno-miR-877 | 0.433845 | 0.202324 | rno-miR-381 | 0.475254 | -0.36352 | rno-miR-3594-5p | 0.450656 | -0.07732 | rno-miR-146a | 0.431444 | 0.099692 |
| rno-miR-496 | 0.509238 | -0.14028 | rno-miR-483 | 0.448204 | -0.05497 | rno-miR-335 | 0.478711 | -0.18279 | rno-miR-326 | 0.454673 | 0.322698 | rno-miR-487b | 0.434181 | -0.06945 |
| rno-miR-93 | 0.510989 | -0.14358 | rno-miR-100 | 0.453742 | -0.04576 | rno-miR-345-3p | 0.482987 | 0.537136 | rno-miR-299 | 0.479727 | -0.17736 | rno-miR-30c-2 | 0.438074 | -0.40729 |
| rno-miR-377 | 0.514796 | -0.12704 | rno-miR-770 | 0.459561 | -0.41056 | rno-miR-503 | 0.486609 | -0.21534 | rno-miR-3554 | 0.482844 | -0.30415 | rno-miR-3557-3p | 0.440222 | -0.28057 |
| rno-miR-411 | 0.520714 | -0.09968 | rno-miR-145 | 0.469602 | -0.43245 | rno-miR-133b | 0.491305 | -0.15401 | rno-miR-3562 | 0.510461 | -0.09193 | rno-miR-18a | 0.445483 | 0.723578 |
| rno-miR-134 | 0.521333 | 0.227902 | rno-miR-7a-2 | 0.469914 | -0.11636 | rno-miR-7a-2 | 0.50034 | 0.036053 | rno-miR-667 | 0.522102 | 0.095042 | rno-miR-383 | 0.450818 | -0.08239 |
| rno-miR-877 | 0.525829 | 0.153598 | rno-miR-193 | 0.471173 | -0.40582 | rno-miR-375 | 0.528589 | 0.206961 | rno-miR-3582 | 0.534371 | 0.120419 | rno-miR-668 | 0.454287 | -0.24344 |
| rno-miR-494 | 0.537775 | 0.188057 | rno-miR-328b-3p | 0.487018 | -0.26201 | rno-miR-675 | 0.540105 | -0.14169 | rno-miR-23a | 0.54328 | 0.081358 | rno-miR-92b | 0.461188 | -0.05744 |
| rno-miR-770 | 0.537778 | 1.068823 | rno-miR-30b-5p | 0.49693 | -0.0932 | rno-miR-3554 | 0.544987 | 0.20576 | rno-let-7i | 0.545248 | 0.232471 | rno-miR-295 | 0.462676 | 1.994969 |
| rno-miR-3561-3p | 0.541421 | -0.19681 | rno-miR-221 | 0.503014 | -0.06012 | rno-miR-298 | 0.545156 | -0.23232 | rno-miR-106b | 0.547238 | 0.056866 | rno-miR-7b | 0.466255 | 0.259329 |
| rno-miR-376b-3p | 0.544877 | -0.06956 | rno-miR-325-3p | 0.504386 | -0.31385 | rno-miR-505 | 0.553988 | 0.150743 | rno-miR-760-5p | 0.563634 | 0.146229 | rno-miR-494 | 0.473282 | 0.236009 |
| rno-miR-25 | 0.547564 | -0.1387 | rno-miR-24-2 | 0.50583 | 0.231194 | rno-miR-484 | 0.569649 | 0.125711 | rno-miR-381 | 0.582929 | 0.196326 | rno-let-7f-1 | 0.49189 | -0.13599 |
| rno-miR-532-3p | 0.557882 | -0.31192 | rno-miR-323 | 0.511075 | 0.291857 | rno-miR-483 | 0.57132 | -0.16998 | rno-miR-383 | 0.596869 | 0.131491 | rno-miR-675 | 0.507499 | 0.184855 |
| rno-miR-451 | 0.563596 | 0.114886 | rno-miR-379 | 0.517576 | -0.25862 | rno-miR-678 | 0.590424 | -0.14093 | rno-miR-503 | 0.598256 | -0.19316 | rno-miR-487b | 0.508396 | -0.21967 |
| rno-miR-376a | 0.575383 | 0.032155 | rno-miR-322 | 0.525256 | -0.2188 | rno-miR-671 | 0.599982 | -0.17678 | rno-miR-222 | 0.619481 | -0.03129 | rno-miR-125b | 0.510252 | -0.12883 |
| rno-miR-296 | 0.578539 | -0.28686 | rno-miR-152 | 0.534177 | -0.19405 | rno-miR-28 | 0.61597 | 0.197533 | rno-miR-139-3p | 0.62572 | -0.05217 | rno-miR-101a | 0.517121 | -2.56998 |
| rno-miR-143 | 0.588321 | -1.67836 | rno-miR-101a | 0.540828 | -0.07681 | rno-miR-3582 | 0.616968 | 0.152653 | rno-miR-150 | 0.653325 | 0.110405 | rno-miR-29b-2 | 0.51798 | -0.1602 |
| rno-miR-20a | 0.588954 | 0.099489 | rno-miR-27b | 0.555757 | 0.054831 | rno-miR-873 | 0.633367 | 0.19024 | rno-miR-181d | 0.669834 | 0.098167 | rno-miR-425 | 0.536963 | 0.382348 |
| rno-miR-375 | 0.590263 | 0.248635 | rno-miR-758 | 0.584152 | -0.12098 | rno-miR-328a | 0.633826 | 0.079845 | rno-miR-425 | 0.671127 | -0.04553 | rno-miR-1188-5p | 0.538538 | 0.188486 |
| rno-miR-760-5p | 0.595662 | -0.28579 | rno-miR-137 | 0.585526 | 0.061797 | rno-miR-3563-5p | 0.63646 | 0.072683 | rno-miR-191 | 0.691775 | 0.022279 | rno-miR-331 | 0.54547 | -0.07144 |
| rno-miR-192 | 0.598207 | 0.336499 | rno-miR-99a | 0.589524 | -0.03922 | rno-miR-132 | 0.641083 | -0.0262 | rno-miR-23b | 0.69279 | -0.03577 | rno-miR-376c | 0.55799 | -0.20159 |
| rno-miR-370 | 0.598607 | -0.45926 | rno-miR-34b | 0.591646 | 0.065328 | rno-miR-221 | 0.658703 | 0.167418 | rno-miR-500 | 0.721323 | -0.05721 | rno-miR-381 | 0.562641 | 0.633437 |
| rno-miR-376c | 0.602983 | -0.15387 | rno-miR-330 | 0.599545 | -0.17304 | rno-miR-152 | 0.663676 | -6.62382 | rno-miR-195 | 0.722641 | 0.089308 | rno-miR-664-2 | 0.564768 | -0.19458 |
| rno-miR-379 | 0.603407 | 0.100106 | rno-miR-125a-5p | 0.604872 | -0.03309 | rno-miR-323 | 0.664448 | 0.099428 | rno-miR-25 | 0.734682 | 0.072144 | rno-miR-338 | 0.580287 | -0.07827 |
| rno-miR-328b-3p | 0.617262 | 0.095447 | rno-miR-344b-2-3p | 0.611729 | -0.24817 | rno-miR-26b | 0.665167 | -0.04806 | rno-miR-145 | 0.739041 | 0.031274 | rno-miR-672 | 0.584611 | -0.04948 |
| rno-miR-344b-2-3p | 0.617568 | -0.16667 | rno-miR-26a | 0.619568 | 0.027516 | rno-miR-344b-1-3p | 0.670561 | 0.246132 | rno-miR-455 | 0.744858 | 0.028004 | rno-miR-504 | 0.590984 | -0.12759 |
| rno-miR-500 | 0.632883 | 0.139726 | rno-miR-339-5p | 0.622082 | -0.197 | rno-miR-19b-1 | 0.673488 | 5.111016 | rno-miR-3120 | 0.750373 | -0.0733 | rno-miR-16 | 0.591569 | 0.089236 |
| rno-miR-872 | 0.659568 | 0.211328 | rno-miR-423 | 0.625935 | 0.144666 | rno-miR-195 | 0.696806 | 0.111435 | rno-miR-31 | 0.764104 | 0.014441 | rno-miR-29a | 0.592282 | -0.04276 |
| rno-miR-423 | 0.660308 | 0.087765 | rno-miR-350 | 0.652027 | 0.092548 | rno-miR-92b | 0.705204 | -1.31636 | rno-miR-431 | 0.77157 | -0.02987 | rno-miR-344b-1-3p | 0.614885 | -0.11039 |
| rno-miR-132 | 0.669491 | 0.040153 | rno-miR-1188-3p | 0.666984 | -0.07064 | rno-miR-329 | 0.715388 | -0.02088 | rno-miR-29c | 0.772466 | 0.026346 | rno-miR-25 | 0.621032 | -0.12626 |
| rno-miR-128 | 0.68745 | 0.031138 | rno-miR-3583-3p | 0.671435 | -1.25064 | rno-miR-143 | 0.724707 | -0.03205 | rno-miR-337 | 0.804048 | 0.026714 | rno-miR-547 | 0.632498 | -0.12384 |
| rno-miR-191 | 0.691694 | 0.297838 | rno-miR-466c | 0.674861 | 0.025502 | rno-miR-30e | 0.753566 | 0.334649 | rno-miR-711 | 0.831299 | -0.07705 | rno-miR-671 | 0.646865 | -0.36411 |
| rno-miR-148b-3p | 0.693534 | -0.06654 | rno-miR-103 | 0.698346 | -0.02561 | rno-miR-511 | 0.766685 | -0.0833 | rno-miR-342-5p | 0.838974 | 0.031051 | rno-miR-100 | 0.670428 | 0.049052 |
| rno-miR-3554 | 0.710178 | -0.10433 | rno-miR-150 | 0.70088 | 0.096921 | rno-miR-193 | 0.776748 | 0.166242 | rno-miR-222 | 0.860222 | -0.3463 | rno-miR-151 | 0.709249 | 0.063873 |
| rno-miR-327 | 0.718204 | 0.061903 | rno-miR-3562 | 0.701942 | 0.069418 | rno-miR-3551-5p | 0.777893 | 4.455202 | rno-miR-224 | 0.861201 | 2.041686 | rno-miR-324-5p | 0.714447 | 0.054072 |
| rno-miR-127 | 0.7208 | -0.06885 | rno-miR-500 | 0.705618 | 0.01939 | rno-miR-3584-3p | 0.778317 | -0.04942 | rno-miR-294 | 0.876133 | -0.01649 | rno-miR-511 | 0.726099 | 0.126766 |
| rno-miR-351 | 0.7522 | -1.16334 | rno-miR-879 | 0.712022 | -0.05195 | rno-miR-25 | 0.786887 | 0.059597 | rno-miR-504 | 0.912876 | -0.00594 | rno-miR-134 | 0.73345 | 0.068004 |
| rno-miR-139-5p | 0.753523 | -0.01393 | rno-miR-702-5p | 0.721977 | 0.059062 | rno-miR-758 | 0.803767 | 0.07367 | rno-let-7b | 0.925351 | 0.010064 | rno-miR-344b-2-3p | 0.734987 | 0.147785 |
| rno-miR-107 | 0.767958 | -0.68532 | rno-miR-380 | 0.730811 | -0.09517 | rno-miR-702-5p | 0.806963 | -0.03731 | rno-miR-598-3p | 0.943816 | -0.00445 | rno-miR-3572 | 0.74362 | 2.267542 |
| rno-miR-3556b | 0.774277 | -0.20545 | rno-miR-28 | 0.735162 | 0.047973 | rno-miR-770 | 0.819489 | 0.044575 | rno-miR-770 | 0.947386 | -0.01397 | rno-miR-770 | 0.749969 | 0.119833 |
| rno-miR-150 | 0.794765 | -0.12841 | rno-miR-134 | 0.746586 | -0.03813 | rno-miR-206 | 0.837071 | -0.96068 | rno-miR-127 | 0.954091 | -0.0084 | rno-miR-339-5p | 0.750831 | 0.192165 |
| rno-let-7c-1 | 0.822799 | -0.27646 | rno-miR-25 | 0.752976 | 0.075801 | rno-miR-292-5p | 0.908804 | -0.00726 | rno-miR-103-1 | 0.958782 | -0.03832 | rno-miR-3596a | 0.755576 | 0.19129 |
| rno-miR-3584-3p | 0.831917 | -0.06934 | rno-miR-23a | 0.75646 | 0.037824 | rno-miR-3085 | 0.917536 | 0.085143 | rno-let-7a | 0.964481 | 0.003821 | rno-miR-3120 | 0.78113 | -0.06674 |
| rno-miR-347 | 0.835081 | -0.31056 | rno-miR-20b-5p | 0.762158 | 0.154081 | rno-miR-674-3p | 0.945729 | -0.00614 | rno-miR-130a | 0.976062 | 0.00108 | rno-miR-92b | 0.794717 | 0.060042 |
| rno-miR-27a | 0.849407 | -0.70494 | rno-miR-30b-3p | 0.76945 | 0.051432 | rno-miR-294 | 0.952824 | 0.480439 |  |  |  | rno-miR-505 | 0.813286 | 0.063163 |
| rno-miR-181c | 0.868907 | 0.061514 | rno-miR-324-5p | 0.77141 | -0.09521 | rno-miR-31 | 0.958495 | 2.930812 |  |  |  | rno-miR-503 | 0.820545 | 0.134294 |
| rno-let-7f-1 | 0.874951 | -0.00057 | rno-miR-301a | 0.771643 | -0.2411 | rno-miR-338 | 0.959971 | -0.00393 |  |  |  | rno-miR-361 | 0.823307 | -0.01453 |
| rno-miR-195 | 0.881308 | -0.03582 | rno-miR-29b-2 | 0.789024 | -0.05573 | rno-miR-299 | 0.963254 | 0.033856 |  |  |  | rno-miR-103 | 0.824033 | -0.01627 |
| rno-miR-764 | 0.883299 | -0.04375 | rno-miR-23b | 0.798225 | -0.0214 | rno-miR-711 | 0.972967 | 0.157443 |  |  |  | rno-miR-3563-3p | 0.837882 | 0.236717 |
| rno-miR-485 | 0.893779 | -0.00691 | rno-miR-30c-1 | 0.83397 | -0.10692 | rno-miR-539 | 0.986135 | -0.0009 |  |  |  | rno-miR-23b | 0.85395 | 0.163789 |
| rno-miR-103 | 0.895446 | -0.11195 | rno-miR-668 | 0.835097 | -0.0877 | rno-miR-377 | 0.999686 | -0.84109 |  |  |  | rno-miR-3558-3p | 0.879598 | -0.07654 |
| rno-miR-341 | 0.902288 | 0.009601 | rno-miR-342-5p | 0.83734 | -0.02682 |  |  |  |  |  |  | rno-miR-137 | 0.882051 | 0.037036 |
| rno-miR-340-5p | 0.902381 | -0.06661 | rno-miR-92b | 0.849918 | -0.1023 |  |  |  |  |  |  | rno-miR-99b | 0.89732 | 0.081621 |
| rno-miR-433 | 0.907103 | -0.37118 | rno-miR-106b | 0.851363 | -0.38683 |  |  |  |  |  |  | rno-miR-133a | 0.913394 | 0.0003 |
| rno-miR-337 | 0.910649 | -0.18235 | rno-miR-147 | 0.853356 | -2.52625 |  |  |  |  |  |  | rno-miR-448 | 0.923741 | 0.049654 |
| rno-miR-23a | 0.914254 | 0.055217 | rno-miR-93 | 0.884266 | 0.051909 |  |  |  |  |  |  | rno-miR-326 | 0.92546 | 0.0278 |
| rno-miR-375 | 0.927783 | 0.016013 | rno-miR-873 | 0.886292 | -0.04587 |  |  |  |  |  |  | rno-miR-540 | 0.934812 | 0.091947 |
| rno-miR-490 | 0.931733 | -0.34028 | rno-miR-33 | 0.887654 | -1.01605 |  |  |  |  |  |  | rno-miR-181d | 0.953426 | -0.03736 |
| rno-miR-365 | 0.938788 | -1.3778 | rno-miR-466b-2 | 0.892388 | 0.007712 |  |  |  |  |  |  | rno-miR-672 | 0.974354 | -0.00215 |
| rno-miR-3563-3p | 0.940768 | -0.11881 | rno-miR-711 | 0.90365 | -0.02371 |  |  |  |  |  |  | rno-miR-675 | 0.975508 | 0.08227 |
| rno-miR-598-5p | 0.944538 | -0.3222 | rno-miR-23a | 0.904218 | 0.076987 |  |  |  |  |  |  | rno-miR-191 | 0.989941 | 0.001682 |
| rno-miR-133b | 0.949656 | -0.00684 | rno-miR-107 | 0.916863 | 0.00643 |  |  |  |  |  |  | rno-miR-195 | 0.994063 | 0.076738 |
| rno-miR-376c | 0.953614 | -0.12446 | rno-miR-874 | 0.92158 | 0.033292 |  |  |  |  |  |  |  |  |  |
| rno-miR-298 | 0.975554 | 0.03738 | rno-miR-664-1 | 0.92585 | 0.06068 |  |  |  |  |  |  |  |  |  |
| rno-miR-344b-1-3p | 0.976698 | 0.059208 | rno-miR-181d | 0.937579 | -0.09358 |  |  |  |  |  |  |  |  |  |
| rno-miR-330 | 0.979176 | -0.14961 | rno-miR-129 | 0.948507 | -0.00529 |  |  |  |  |  |  |  |  |  |
| rno-miR-3596a | 0.989275 | -3.06427 | rno-miR-466b-1 | 0.950083 | -0.00584 |  |  |  |  |  |  |  |  |  |
| rno-miR-323 | 0.993253 | 0.02446 | rno-miR-130a | 0.950552 | -0.00443 |  |  |  |  |  |  |  |  |  |
|  |  |  | rno-miR-7a-1 | 0.956823 | 0.034832 |  |  |  |  |  |  |  |  |  |
|  |  |  | rno-miR-211 | 0.974065 | -0.03849 |  |  |  |  |  |  |  |  |  |
|  |  |  | rno-miR-34c | 0.986396 | -0.02505 |  |  |  |  |  |  |  |  |  |
|  |  |  | rno-miR-16 | 0.99148 | -0.17073 |  |  |  |  |  |  |  |  |  |
